# Sequence‐encoded determinants drive distinct early aggregation pathways and different structural outcomes in PNT1 fibrils across *Henipavirus* members

**DOI:** 10.1002/pro.70758

**Published:** 2026-08-11

**Authors:** Harshita Sawdekar, Julien Mignon, Frank Gondelaud, Anuja Walimbe, Samrat Mukhopadhyay, Joseph Chamieh, Sonia Longhi

**Affiliations:** ^1^ Aix‐Marseille Université, CNRS, Architecture et Fonction des Macromolécules Biologiques (AFMB), UMR 7257 Marseille France; ^2^ IBMM, University of Montpellier, CNRS, ENSCM Montpellier France; ^3^ Centre for Protein Science, Design, and Engineering Indian Institute of Science Education and Research (IISER) Mohali Punjab India; ^4^ Department of Chemical Sciences Indian Institute of Science Education and Research (IISER) Mohali Punjab India; ^5^ Department of Biological Sciences Indian Institute of Science Education and Research (IISER) Mohali Punjab India

**Keywords:** aggregation, aggregation‐prone regions, amyloidogenic region/motifs, fibrillation, molecular dynamics, Raman spectroscopy, Taylor dispersion analysis

## Abstract

Protein aggregation is increasingly recognized as a biologically relevant process in viral proteins, yet the molecular determinants governing such phenomena remain poorly understood. Intrinsically disordered proteins (IDPs) are ubiquitous in viral proteomes, where conformational plasticity not only enables functional diversity but also permits aberrant self‐assembly. Members of the *Paramyxoviridae* family, including henipaviruses such as the Nipah and Hendra viruses (two biosafety level‐4 pathogens), encode the P, V, and W proteins that share a common intrinsically disordered N‐terminal domain (NTD). V and W are virulence factors that undergo fibrillation. Here, we explored the aggregation landscape of seven homologous PNT1 subdomains from the NTD that harbors a conserved cryptic amyloidogenic region (CAR). By integrating Taylor dispersion analysis (TDA), Raman spectroscopy, and all‐atom molecular dynamics (MD) simulations, we unraveled both early assembly kinetics and structural outcomes. Despite the conserved CAR motif, the PNT1 homologs exhibit strikingly divergent aggregation behaviors. TDA revealed distinct oligomerization pathways with variations in nucleation, growth kinetics, and monomer recruitment, indicating virus‐specific pathways. Raman spectroscopy unveiled substantial variations in β‐sheet content, side‐chain packing, and aromatic residue environments, while MD simulations showed that conformational heterogeneity in the CAR flanking regions modulates hydrogen‐bond networks and structural stability. These findings indicate that the sequence context beyond a conserved amyloidogenic core governs the aggregation landscape of *Henipavirus* P, V, and W proteins. Evolutionary diversification modulates conformational heterogeneity and assembly pathways, giving rise to distinct structural outcomes. This work sheds light on the molecular grammar underlying viral protein fibrillation with potential implications for viral pathogenicity.

## 1 INTRODUCTION

Over recent years, protein misfolding and fibrillation have emerged as recurring molecular themes in virology, with implications for viral replication, adaptation to the host, and disease severity (Gondelaud et al., 2023). A large fraction of aggregation‐prone proteins belongs to the class of intrinsically disordered proteins (IDPs), which do not adopt a single stable conformation but instead populate dynamic ensembles across a relatively flat and frustrated energy landscape (Habchi et al., 2010; Uversky, 2014). This intrinsic plasticity enables IDPs to access higher‐order assemblies, including amyloid fibrils characterized by a cross‐β architecture in which β‐strands are oriented perpendicular to the fibril axis. Notably, such supramolecular assemblies are not restricted to pathological contexts but are observed across diverse biological systems, from unicellular organisms to higher eukaryotes, underscoring their fundamental physicochemical basis (Buchanan et al., 2023).

IDPs and intrinsically disordered regions (IDRs) are now recognized as central components of viral proteomes, where conformational flexibility enables compact genomes to encode functional diversity, binding promiscuity, and regulatory adaptability without reliance on extensive folded domains (Xue et al., 2014). This paradigm is particularly relevant to *Paramyxoviridae* (Erales et al., 2015; Longhi et al., 2017), a family of negative‐sense, single‐stranded RNA viruses within the order *Mononegavirales*, which includes clinically significant pathogens, such as measles, mumps, and parainfluenza viruses, as well as the Nipah and Hendra henipaviruses. In these viruses, various proteins exhibit an intrinsic propensity to assemble into higher‐order fibrillar structures, suggesting that disorder‐to‐aggregate transitions may represent an underappreciated facet of viral molecular biology (Gondelaud et al., 2026; Gondelaud, Bignon, et al., 2025; Gondelaud, Leval, et al., 2025; Nilsson et al., 2022; Pesce et al., 2022; Pesce et al., 2025; Salladini et al. 2021a). Despite growing evidence for such structural plasticity, the sequence‐level determinants that govern viral protein aggregation and the evolutionary pressures shaping these behaviors remain poorly understood.

Henipaviruses pose a significant global health threat (Quarleri et al., 2022). Nipah and Hendra viruses (NiV and HeV) are classified as biosafety level‐4  pathogens and are associated with severe respiratory and/or encephalitic diseases in humans and animals, with case fatality rates often exceeding 70% (Kaza & Aguilar, 2023; Li et al., 2023). Recurrent zoonotic spillover events from bat reservoirs to livestock and human populations have been documented across Southeast Asia and Australia (Kaza & Aguilar, 2023). Beyond their impact on human health, NiV and HeV outbreaks have substantial economic and ecological consequences. These observations underscore the importance of understanding the molecular determinants that underlie viral pathogenicity and adaptation to the host.

**FIGURE 1:**
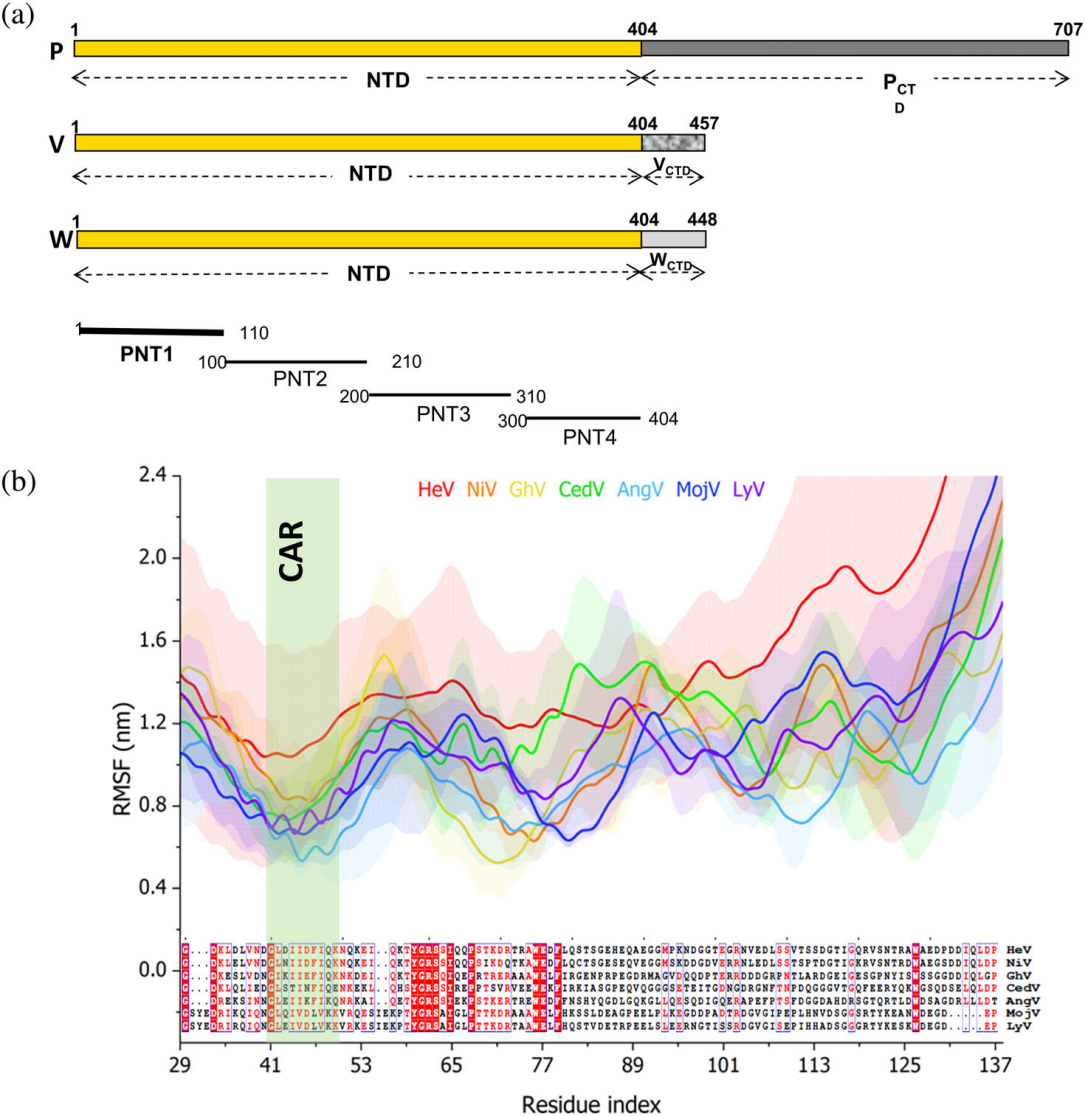
Organization of the Henipavirus P, V, and W proteins and sequence conservation along with conformational dynamics of the PNT1 subdomain across seven representative Henipavirus members. (a) Schematic organization of the Henipavirus P, V and W proteins generated by RNA editing, highlighting the shared intrinsically disordered N‐terminal domain (NTD; yellow), subdivided into the PNT1, PNT2, PNT3, and PNT4 regions. Sequence numbering refers to the HeV proteins. (b) Time‐averaged root‐mean‐square fluctuation (RMSF) along the sequence of the PNT1 subdomain of HeV (red), NiV (orange), GhV (yellow), CedV (green), AngV (blue), MojV (indigo), and LyV (violet) simulated for 1 μs. Curves correspond to the average of triplicates with the standard deviation represented as a trace (shaded area) in the virus‐associated color. The CAR motif is highlighted in mint green. Sequence alignment of the first 110 amino acids (PNT1 subdomain) of the seven major Henipavirus P/V/W proteins, along with the derived consensus sequence, as obtained using ClustalW and ESPript. Residues corresponding to a similarity above 60% are boxed and shown in red. Identical residues are boxed and shown in white on a red background. Dots above the alignment indicate intervals of 10 residues. Numbering starts at residue 29; residues 1–28 originate from the expression vector.

Recent studies have highlighted that the P, V, and W proteins from NiV and HeV contain extensive IDRs capable of undergoing disorder‐to‐aggregate transitions. The latter raises the possibility that viral protein fibrillation directly contributes to cellular dysfunction during infection. In neuronal contexts, aggregation of viral proteins may disrupt intracellular organization, promote inclusion body formation, and exacerbate inflammatory responses associated with viral encephalitis. However, despite these emerging insights, the molecular grammar that governs self‐assembly within paramyxoviral proteins remains largely unresolved, which hinders our understanding of the underlying pathogenic mechanisms, thus limiting the design of rational antiviral approaches. In many *Paramyxoviridae* members, including NiV and HeV, the genome encodes multiple proteins from a limited number of transcriptional units, with the P gene serving as a prominent example of functional diversification. Through mRNA editing and alternative reading frames, this gene gives rise to the P, V, W, and C proteins. The P, V, and W proteins share an intrinsically disordered N‐terminal domain (NTD) but diverge in their C‐terminal regions, enabling distinct functional roles (Figure 1a). While the P protein plays a pivotal role in transcription and replication, enabling recruitment of the viral polymerase onto the nucleocapsid template and preventing self‐assembly of the N protein in the absence of ongoing RNA synthesis (Bloyet, 2021), the V and W proteins act as potent antagonists of host innate immunity. Although both proteins suppress type I interferon signaling by preventing STAT1 phosphorylation through their shared intrinsically disordered NTD, they employ distinct mechanisms: the V protein primarily targets cytoplasmic antiviral signaling pathways, including RIG‐I/MDA5 and STAT activation, whereas the W protein accumulates in the nucleus, where it suppresses interferon and inflammatory signaling through inhibition of IRF3 and sequestration of STAT1 (Shaw et al., 2004; Tripp et al., 2025). In addition, the C‐terminal region of the W proteins binds to 14‐3‐3 proteins, leading to accumulation of the latter in the nucleus. This accumulation enhances the nuclear export of p65, reducing the ability of the latter to interact with its promoter targets, thereby inhibiting the NF‐κB‐induced proinflammatory response (Enchéry et al., 2021). Recent work further demonstrated that the redox‐dependent condensation of the W protein directly modulates its ability to regulate type I IFN and NF‐κB signaling, establishing a mechanistic link between the biophysical properties of the shared disordered NTD and innate immune response evasion (Gondelaud et al., 2026).

Within the disordered NTD of the HeV V protein, a region (referred to as PNT3, a.a. 200 to 310) (Figure 1a) was found to undergo a liquid‐to‐hydrogel phase transition (Salladini et al. 2021a). PNT3 was shown to also be able to assemble into amyloid‐like fibrils (Gondelaud, Leval, et al., 2025; Nilsson et al., 2022; Salladini et al., 2021b). The W protein exhibits a similar behavior, forming fibrillar assemblies both in vitro (Pesce et al., 2022, 2025) and within the nuclei of transfected cells (Gondelaud et al., 2026), pointing to a potential functional role in viral pathogenesis and, in particular, in host innate immunity evasion. Previous work established that the PNT1 region (residues 1–110) (Figure 1a) is the primary driver of HeV W fibrillation, identifying a conserved cryptic amyloidogenic region (CAR, a.a. 10 to 19) as a core aggregation‐prone segment whose nucleation is modulated by adjacent fluctuating α‐helices (Gondelaud, Bignon, et al., 2025). While residues 2–9 are dispensable, residues 20–29 act synergistically to promote nucleation. Transient α‐helical elements are partially populated in both unassembled and assembled states, whereas β‐strands are stabilized as β‐sheet cores within fibrils (Gondelaud, Bignon, et al., 2025). Sequence alignment revealed that the CAR is highly conserved across *Henipavirus* species, suggesting a fundamental role in viral molecular function and potentially in pathogenesis. Consistent with the high conservation of this CAR across *Henipavirus* species, the ability to form fibrils was shown to be conserved across PNT1 domains of other henipaviruses, highlighting a shared molecular feature within the genus (Gondelaud, Bignon, et al., 2025). Phylogenetic analyses indicate that modern henipaviruses diverged from a common ancestor while adapting to distinct ecological niches, particularly within bat reservoirs (Kane et al., 2025). Such divergence raises the possibility that viral evolution has modulated the aggregation propensities of these proteins, with potential consequences for transmissibility, host range, and pathogenicity. In this context, *henipavirus* PNT1 domains represent compelling systems for investigating how sequence variation encodes functional and structural diversity within a conserved aggregation‐prone framework.

In this study, we investigated the mechanisms underlying fibrillation of PNT1 across multiple henipaviruses, namely the HeV, NiV, Ghana (GhV), Cedar (CedV), Angavokely (AngV), Mòjiāng (MojV), and Langya (LyV) viruses. While previous studies contributed to unveiling the molecular grammar of HeV PNT1 fibrillation (Gondelaud, Bignon, et al., 2025), the kinetics of the process, as well as possible subtle differences among PNT1 subdomains from different *Henipavirus* members, remained to be unraveled. To shed light on these hitherto unexplored aspects, we herein employed a multimodal biophysical approach integrating Taylor dispersion analysis (TDA), Raman spectroscopy, and all‐atom molecular dynamics (MD) simulations. This combination enabled high‐resolution characterization of aggregation kinetics, structural transitions, and conformational landscapes, including the detection of transient intermediates that are otherwise inaccessible to ensemble‐averaged techniques.

Results showed that the sequence context outside the conserved amyloidogenic core dictates the pathway and structural outcome of protein aggregation. Notwithstanding a shared fibrillogenic motif, the viral proteins herein studied were found to access distinct aggregation pathways and produce structurally diverse assemblies. Our findings reveal that viral evolution has diversified the molecular grammar of amyloid formation within henipaviruses, with potential implications for viral biology and disease mechanisms.

## 2 RESULTS & DISCUSSION

### 2.1 Sequence conservation and protein flexibility along the seven PNT1 sequences

We first performed a comparative bioinformatic and dynamic analysis of the seven PNT1 subdomains. In accordance with previous studies (Gondelaud, Bignon, et al., 2025), multiple sequence alignment revealed a highly conserved segment falling within the CAR. Residues with >60% similarity or identity cluster within this motif, consistent with strong evolutionary constraints (Figure 1b).

To probe the dynamic basis of this conservation, we quantified backbone flexibility using root‐mean‐square fluctuation (RMSF) profiles averaged over triplicated all‐atom MD simulations. Although flexibility varies across most of the sequence, all proteins exhibit a pronounced and conserved RMSF minimum that precisely localizes to the CAR motif (Figure 1b), identifying it as the least flexible region across all PNT1 homologs. Such reduced backbone mobility is indicative of a shared structural constraint and supports a functional role for the CAR as a nucleating element for aggregation. Its relative rigidity likely facilitates intermolecular recognition and stabilizes early assembly interfaces, thereby promoting fibril formation. Notably, a second region of reduced flexibility is observed between residues 65–77, corresponding to the nucleoprotein‐binding site of the P protein (Longhi et al., 2017). The conservation of restricted dynamics within this segment is consistent with the critical role of N–P interactions in the viral replication cycle, suggesting that evolutionary pressures preserve not only sequence identity but also the dynamic properties required for function.

By contrast, regions flanking the CAR display greater and more heterogeneous flexibility, particularly toward the C‐terminus (Figure 1b). These sequence‐dependent differences in dynamics suggest that, while the CAR motif defines a common aggregation nucleus, surrounding regions modulate the accessibility and stabilization of aggregation‐prone intermediates. Together, these results support a model in which a conserved and rigid amyloidogenic core is embedded within more flexible sequence stretches that contextually may tune aggregation pathways and structural outcomes among PNT1 variants.

### 2.2 Divergent early aggregation profiles revealed by Taylor dispersion analysis

One of the main objectives of this work was to compare the aggregation kinetics of the PNT1 subdomain across seven *Henipavirus* members. Because they share a conserved amyloidogenic core and similar disorder propensities (Gondelaud, Bignon, et al., 2025), these sequences provide a controlled framework to assess how sequence context modulates early self‐assembly. In order to document the early aggregation events, we used TDA. In contrast to bulk fluorescence or turbidity measurements, TDA is sensitive to transient oligomeric intermediates and enables discrimination of parallel aggregation pathways that are otherwise obscured in ensemble‐averaged assays (Deleanu et al., 2021). TDA thus gives access to the distributions of different species present during the aggregation process in terms of hydrodynamic radii (*R*_*h*_) and proportions in solution as a function of aggregation time.

**FIGURE 2:**
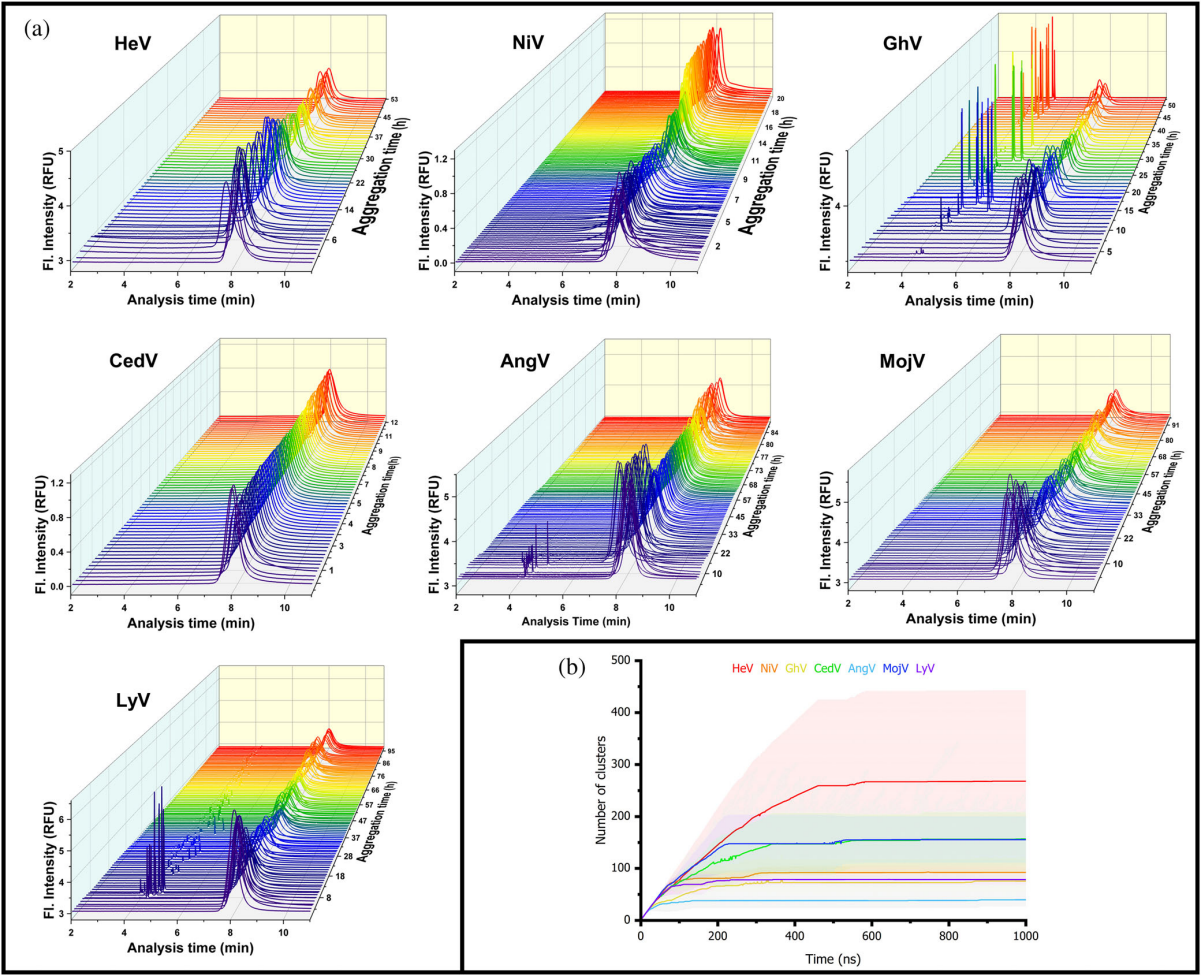
Aggregation behavior and conformational clustering of the PNT1 subdomain across representative Henipavirus homologs. Evolution of the elution profiles obtained from TDA over the course of aggregation for (a) HeV, NiV, GhV, CedV, AngV, MojV, and LyV PNT1. All analyses were performed with 50 μM protein in 20 mM HEPES, 150 mM NaCl, and 0.5 M urea at 37°C, pH 7.2. Fused silica capillaries: 49.6 μm i.d. × 60 cm × 39 cm. Mobilization pressure: 50 mbar. Injection: 30 mbar for 6 s, Vi ≈5.6 nL (Vi/Vd ≤1%). Fluorescence detection at 275 nm. (h) Time evolution of the number of clusters defined with a 5 Å RMSD cutoff for the PNT1 subdomain of HeV (red), NiV (orange), GhV (yellow), CedV (green), AngV (blue), MojV (indigo), and LyV (violet) simulated for 1 μs. Curves correspond to the average of triplicates with the standard deviation represented as a trace (shaded area) in the virus‐associated color.

The PNT1 proteins were all purified under denaturing conditions in three steps (Figure S1). For all the seven PNT1 proteins, aggregation was initiated by rapid dilution into identical buffer conditions (Buffer D, see Materials and Methods), resulting in a final protein concentration of 50 μM and a final urea concentration below 0.5 M, a concentration known not to perturb protein folding or assembly (Ahmad et al., 2004; Madani et al., 2025). The three‐dimensional representations of the Taylorgrams (Figure 2a) reveal that despite a shared fibrillogenic core, the seven proteins exhibit distinct early aggregation behaviors. HeV PNT1 shows a rapid increase in signal intensity followed by an exponential decay, indicative of the formation of higher‐order species that are kinetically unstable and undergo dissociation or sedimentation (Figure 2a). This behavior suggests the presence of competing, non‐productive assembly pathways, which may hinder the overall efficiency of the assembly process and lead to the formation of metastable conformers. In contrast, NiV PNT1 displays a gradual evolution in peak shape consistent with the emergence of higher‐mass populations, followed by a steady growth toward a saturation regime, indicative of a more controlled assembly pathway (Figure 2a). CedV PNT1 exhibits minimal peak‐shape evolution, with a predominantly monodisperse distribution that can mask low‐abundance oligomeric species, with only subtle changes observed at later time points (>12 h) (Figure 2a). AngV PNT1 displays a delayed transition, characterized by the appearance, within the first ~10 h, of “spikes”, i.e., thin peaks eluting before the main peak, followed by a pronounced decrease in the main peak's intensity after a prolonged incubation (>30 h) (Figure 2a). Spikes are peaks deviating from Gaussian dispersion indicative of non‐dispersive higher‐mass species outside the Taylor regime, which cannot be sized by TDA. The Taylorgrams of AngV PNT1 are thus consistent with delayed aggregation and depletion of soluble species. MojV PNT1 shows a decrease in peak area after about 15 h, along with increased peak broadening, suggesting a lag phase in which monomers formed nuclei that then led to the formation of higher‐order oligomers, which appear to remain unchanged even after 100 h, as the peak shape shows no further evolution after 15 h and no spikes were observed (Figure 2a). Finally, GhV, and LyV PNT1 exhibit similar dissociative tendencies (Figure 2a), where their Taylorgrams display early emergence of sharp, non‐Gaussian spikes (~3 and ~8 h, respectively) corresponding to higher‐order or highly flexible protofibrillar assemblies that fall outside the classical dispersion regime. However, no change in peak broadening was observed, suggesting that the intermediate species rapidly transformed into higher‐order assemblies, with monomers subsequently adding to these formed assemblies, leading to a decrease in peak area. This suggests a secondary nucleation mechanism in which monomers add to already‐formed fibers.

The evolution of conformational heterogeneity, quantified by the number of structural clusters sampled over the course of MD simulations, is consistent with the aggregation pathways described by TDA (Figure 2b). All proteins show an initial increase in cluster number, reflecting early diversification of conformational states. However, the extent and progression of this heterogeneity differ markedly. As shown by the plateau of clustering curves, every PNT1 subdomain tends to stabilize into a homogeneous conformational ensemble within the first half of the simulation. Such profiles arguably arise from restricted backbone motions due to the ordering of the initially disordered chains. Notwithstanding such a converging property, heightened deviation is observed for CedV, MojV, and most especially HeV PNT1, hence reflecting increased structural heterogeneity between the nearest neighbors. In other words, CedV, MojV, and HeV PNT1 appear to have greater chain dynamics so that the search for a metastable conformer takes longer than the other PNT1 proteins. In contrast, NiV and AngV PNT1 reach an early plateau with a limited number of clusters, indicative of a restricted conformational landscape consistent with a more equilibrium‐governed assembly pathway. GhV and LyV PNT1 exhibit moderate cluster growth, indicative of intermediate heterogeneity and seemingly consistent with non‐classical aggregation pathways involving higher‐order or protofibrillar species.

Collectively, these results establish a direct relationship between variant‐dependent conformational heterogeneity and the diversity of aggregation pathways. Variations in structural sampling govern the stability, kinetics, and mechanism of early assembly, demonstrating that closely related viral proteins with conserved fibrillogenic cores can access distinct aggregation trajectories under identical conditions. These findings highlight the dominant role of sequence context, particularly the regions flanking the amyloidogenic core, in shaping early aggregation behavior and determining pathway selection.

### 2.3 Evolution of peak area and hydrodynamic radius during aggregation kinetics

**FIGURE 3:**
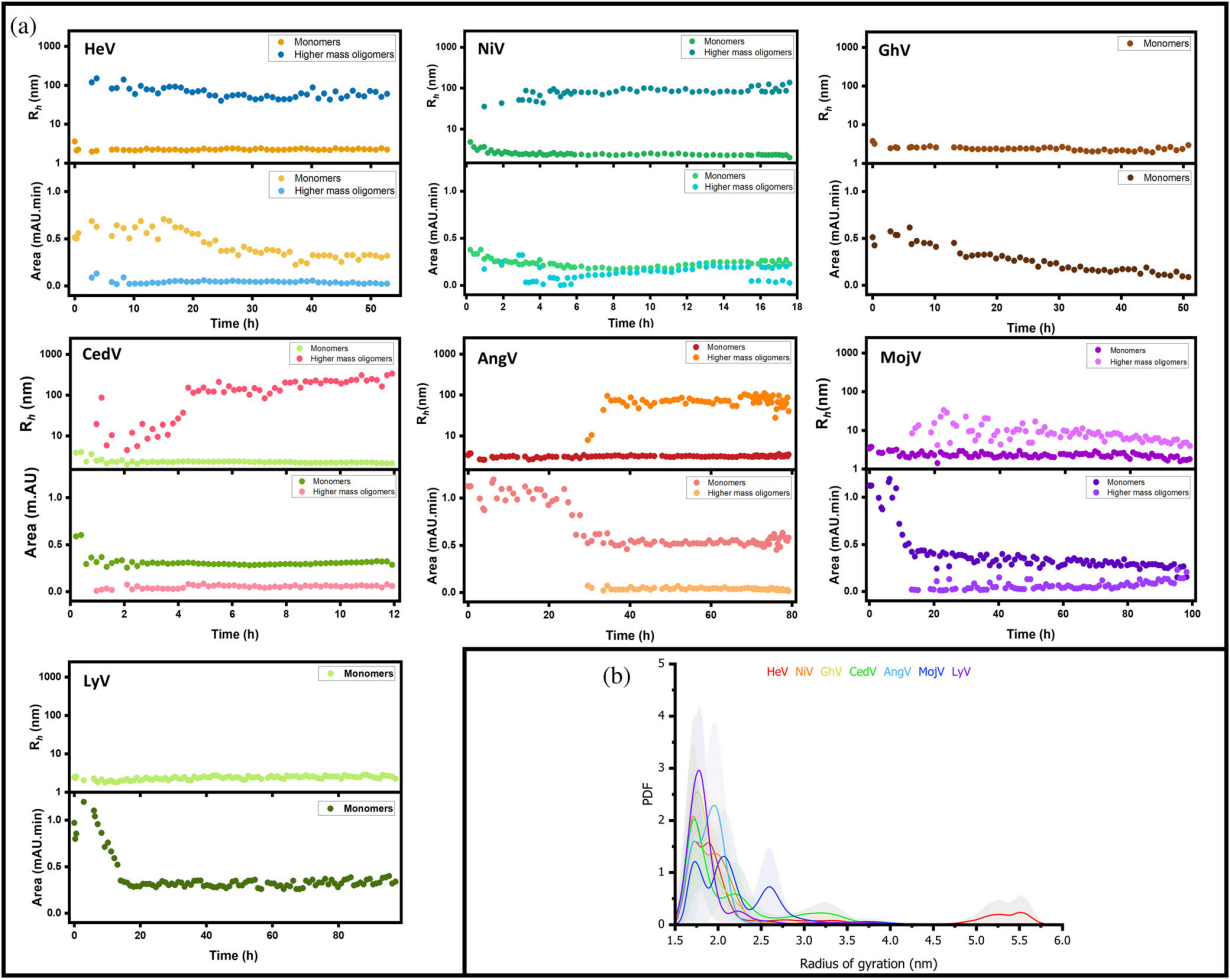
Hydrodynamic expansion and molecular compactness of PNT1 subdomains during aggregation. Evolution of hydrodynamic radius (in nm) (Rh, top) and peak area (in mAU·min) (bottom) over the course of aggregation for monomeric and higher‐mass species as obtained by Taylor dispersion analysis for (a) HeV, NiV, GhV, CedV, MojV, AngV and LyV PNT1. (b) Distribution of the radius of gyration (*R*_*g*_), defined as a probability density function (PDF), for the PNT1 subdomain of HeV (red), NiV (orange), GhV (yellow), CedV (green), AngV (blue), MojV (indigo), and LyV (violet) simulated for 1 μs. Curves correspond to the average of triplicates with the standard deviation represented as a trace (shaded area) in the virus‐associated color.

**FIGURE 4:**
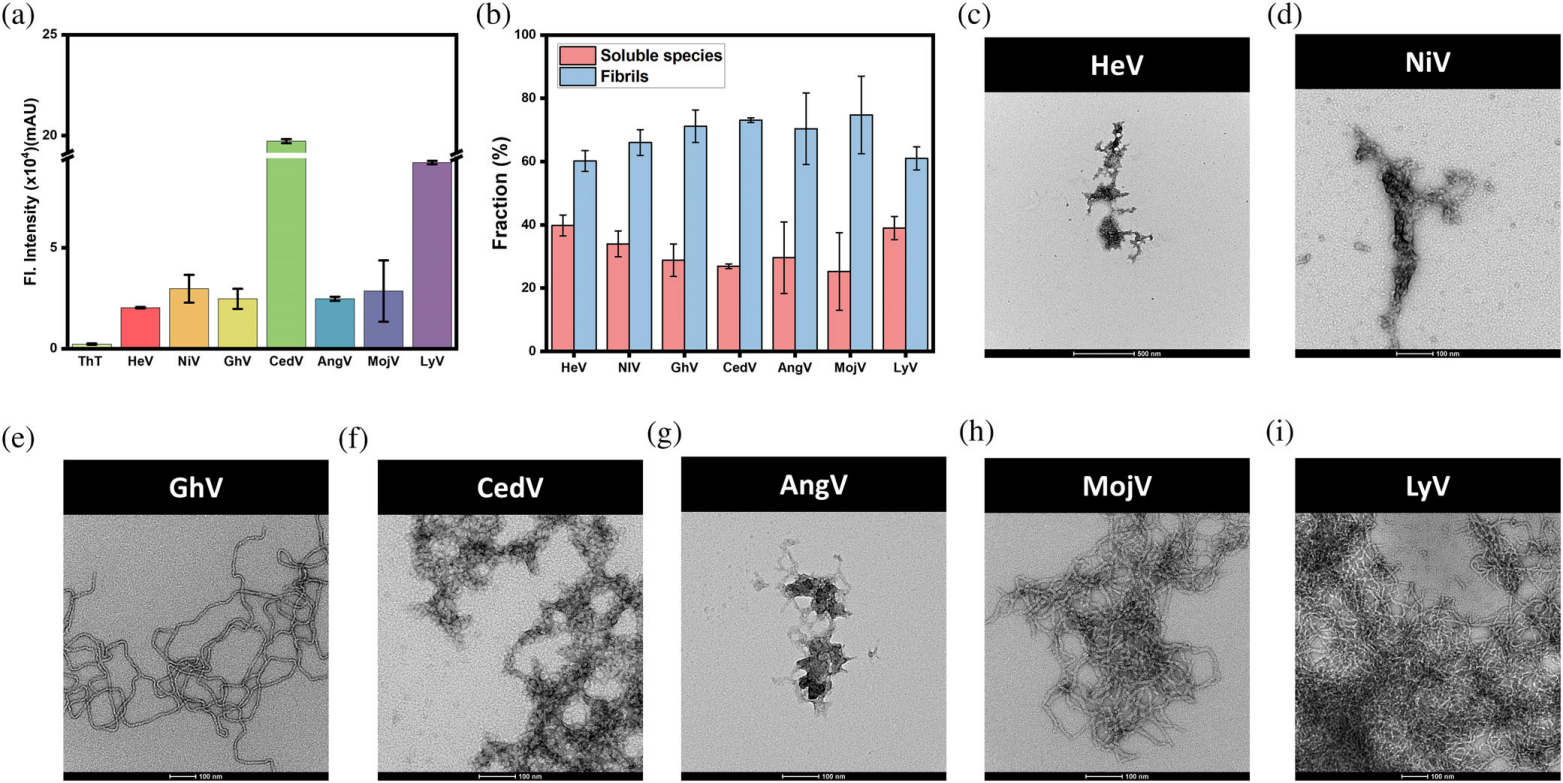
(a) ThT fluorescence intensity recorded for all the seven proteins after 100 h of incubation in 20 mM HEPES, 150 mM NaCl, pH 7.2; ThT alone was incubated as a control. All protein samples exhibit increased fluorescence relative to ThT alone, indicating binding of ThT to β‐sheet‐rich amyloid structures formed during incubation. Differences in fluorescence intensity reflect variability in fibril formation and amyloid content across the homologs. All protein samples were incubated at 37°C. Data are shown as mean ± SD. (b) Sedimentation assays were used to quantify soluble species and insoluble fibrillar fractions after 100 h of incubation. Soluble species percentage (in light red) was calculated from the protein concentration remaining in the supernatant relative to the initial total concentration, and fibril percentage (in light blue) was determined from the protein concentration of the sedimented fraction (*n* = 9). Ns‐TEM observations of (c) HeV, (d) NiV, (e) GhV, (f) CedV, (g) AngV, (h) MojV, and (i) LyV PNT1 after 100 h of incubation. Scale bar: 100 nm.

To further dissect early assembly behavior, time‐resolved Taylor dispersion profiles were deconvoluted to extract distinct populations with characteristic hydrodynamic radii (Figure 3a). This was achieved by fitting the elution profile with the sum of *n* Gaussian functions. This analysis revealed pronounced divergence in aggregation pathways across the seven PNT1 proteins. Although all proteins ultimately form fibrils (Figure 4), the early‐stage kinetics and intermediate populations differ substantially.

HeV PNT1 shows rapid formation of large species (*R*_*h*_ ≈150 nm) within ~3 h, followed by a progressive decay of their size which stabilizes around *R*_*h*_ ≈60 nm after ~25 h (Figure 3a). This transition coincides with a decrease in monomer population (*R*_*h*_ ≈2.4 nm) beyond ~19 h and reaches a lower plateau around 25 h. In contrast, NiV PNT1 follows an equilibrium‐governed pathway, with oligomeric species (*R*_*h*_ ≈35 nm) emerging within ~1 h alongside a reduction in monomer peak area, consistent with monomer recruitment (Figure 3a). A stable coexistence of monomeric and oligomeric populations is established by ~8 h. For CedV PNT1, deconvolution of the elution profiles revealed the formation of small oligomers (*R*_*h*_ ≈19–20 nm) within ~2 h, followed by their growth after ~4 h (Figure 3a). The concurrent decrease in monomer population indicates incorporation into these early assemblies, which likely serve as nucleation‐competent intermediates that promote continued aggregation. AngV PNT1 exhibits delayed aggregation, with a sharp increase in oligomer size to about 85–90 nm observed only after >30 h, accompanied by monomer depletion, indicative of a higher kinetic barrier to aggregation (Figure 3a). While the oligomeric state is initially stable, it becomes increasingly transient beyond ~70 h, suggesting reduced stability during progression toward fibril formation. MojV PNT1 forms transient oligomers (*R*_*h*_ ≈33 nm) within ~20 h, accompanied by a decrease in monomer population with no significant evolution in size or proportion even after 100 h (Figure 3a). Nevertheless, negative‐staining transmission electron microscopy (ns‐TEM) confirms fibril formation under identical conditions (Figure 4h). The absence of corresponding high‐R_*h*_ species in TDA suggests that larger assemblies either sediment or fall outside the measurable dispersion regime. Consequently, the deconvoluted profiles primarily capture smaller oligomeric species, while the apparent decrease in *R*_*h*_ likely reflects fragmentation or depletion of larger aggregates. As already discussed, GhV and LyV (Figure 2a) exhibit early non‐Gaussian spikes, indicative of non‐dispersive higher‐order or protofibrillar species accompanied by rapid monomer depletion. These features fall outside the classical Taylor dispersion regime, consistent with the formation of large and anisotropic assemblies not accurately described by diffusion‐based analysis. Ns‐TEM observations at later time points confirm fibril formation (Figure 4e,i), suggesting that these early species rapidly evolve into higher‐order aggregates. Following this initial phase, the Taylorgrams predominantly resolve into monomeric populations despite continued monomer depletion upon deconvolution, indicating sequestration of protein into larger species that escape detection. It is noteworthy that LyV shows a steeper decrease in the monomer population around 15 h, suggesting a catalysis of aggregation via nucleus formation, while GhV shows a continuous decrease of the monomer population, suggesting continuous elongation of the aggregates.

Analysis of peak area evolution further supports these mechanistic distinctions. Proteins exhibiting increasing R_*h*_ show a progressive decrease in the monomer‐associated peak area, consistent with the incorporation into larger assemblies. On the contrary, systems with stable or decreasing R_*h*_ display less pronounced peak area loss, suggesting persistence of smaller oligomeric species or dynamic equilibrium between populations. In some cases, transient increases in peak area accompany fluctuations in R_*h*_, likely reflecting the formation of intermediate oligomeric states prior to further assembly or reorganization. Comparison with MD‐derived radius of gyration (*R*_*g*_) distributions reveals a direct relationship between conformational properties and aggregation behavior (Figure 3b). Each PNT1 protein exhibits a major population confined at a low *R*_*g*_ value of 1.7 nm, which is congruent with the compaction of the protein chain across the tested viral proteins. However, the distribution profiles intriguingly reveal discrepancies, with virus‐specific subpopulations scattered at higher *R*_*g*_ values. For example, NiV PNT1 is only characterized by a second population at around 2.0 nm, while MojV PNT1 samples two additional and well‐defined *R*_*g*_ spaces at 2.1 and 2.6 nm. Notably, HeV PNT1 differs the most by sustaining a subpopulation of highly expanded conformations comprised of between 5.0 and 5.8 nm. Taken together, these results support a scenario where PNT1 subdomains, while converging toward conformers of similar dimensions, follow a variant‐dependent structural collapse and rearrangement pathway, presumably directing their fibrillation mechanism through the formation of distinct early‐stage aggregation intermediates. Proteins exhibiting broader *R*_*g*_ distributions correlate with R_*h*_ experimentally shown to be metastable, indicating that conformational expansion facilitates intermolecular association and aggregate growth. Conversely, proteins with narrower *R*_*g*_ distributions centered at smaller values form more compact oligomers, consistent with limited changes in *R*_*h*_ and reduced aggregation propensity.

Collectively, these findings strongly suggest that aggregation proceeds through multiple distinct pathways rather than through a single unified mechanism. While some variants follow a nucleation–growth pathway characterized by gradual size increase and continuous monomer depletion, others exhibit rapid formation of transient or non‐equilibrium assemblies that bypass stable oligomeric intermediates. These results further confirm that proteins sharing a conserved fibrillogenic core can access diverse aggregation trajectories governed by variant‐dependent conformational landscapes. Variations in oligomer stability, growth kinetics, and pathway selection once more seem to arise from differences in flanking sequence context, which modulate early conformational heterogeneity and thereby dictate the mechanism of self‐assembly.

### 2.4 Raman spectroscopic characterization of PNT1 amyloidogenicity

**FIGURE 5:**
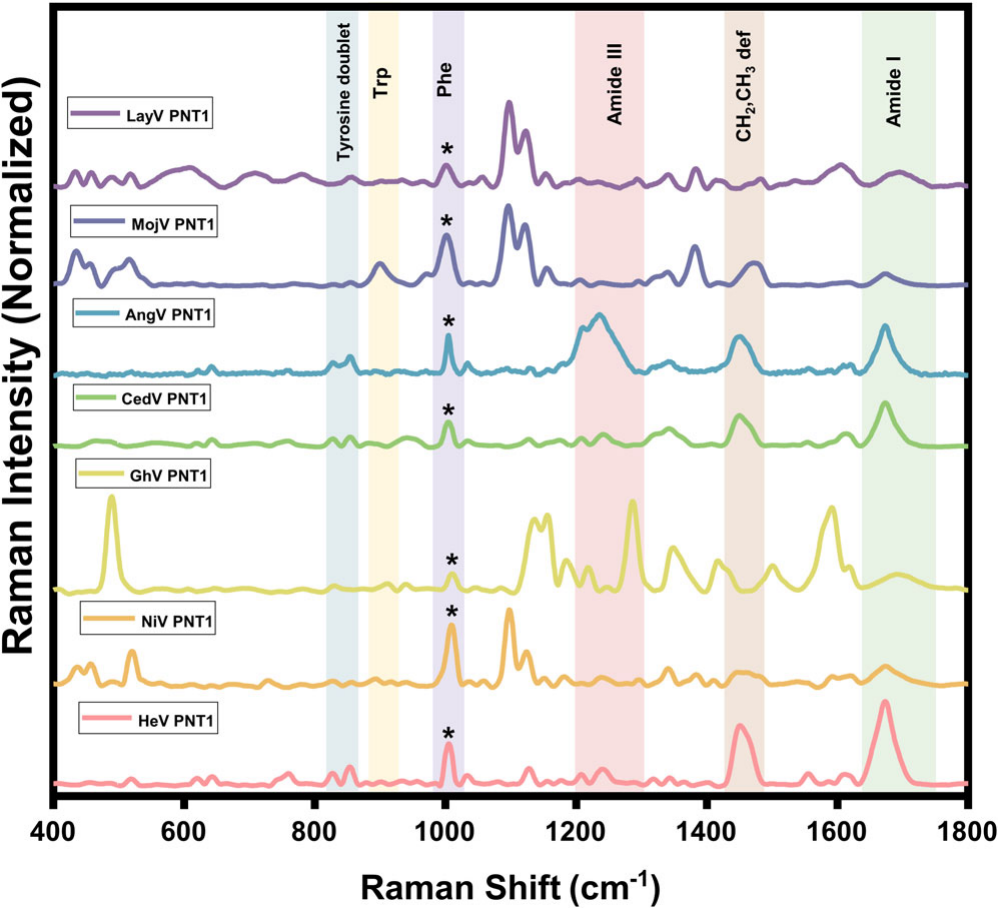
Comparative Raman spectroscopic signatures of *Henipavirus* PNT1 fibrils. Average Raman spectrum of HeV (red), NiV (orange), GhV (yellow), CedV (green), AngV (blue), MojV (indigo), LyV (violet). All spectra are normalized with respect to the phenylalanine ring breathing band at 1005 cm^−1^ marked by an asterisk.

To probe the secondary structure and side‐chain microenvironments at the end stage of aggregation and also to establish the vibrational Raman signature of different assemblies across species, Raman measurements were carried out on mature protein fibrils incubated for 100 h, in the same conditions used for TDA. Figure 5 and Figure S2 show the Raman spectra (400–1800 cm^−1^) of the seven PNT1 homologs. As discussed above, these homologs substantially differ in their aggregation behaviors. Raman analysis provides complementary structural insight into these differences by reporting on backbone conformation (amide I and III regions) and side‐chain packing (aromatic and aliphatic vibrational modes). Amide I and amide III are canonical spectral marker bands for secondary structures (Kuhar et al., 2018; Lippert et al., 1976; Maiti et al., 2004; Rygula et al., 2013; Tuma, 2005). The amide I region (1600–1700 cm^−1^) primarily arises from the C=O stretching vibration of the peptide backbone and is widely used to assess protein secondary structure (Kuhar et al., 2018, Lippert et al., 1976, Maiti et al., 2004, Rygula et al., 2013, Tuma, 2005).

**FIGURE 6:**
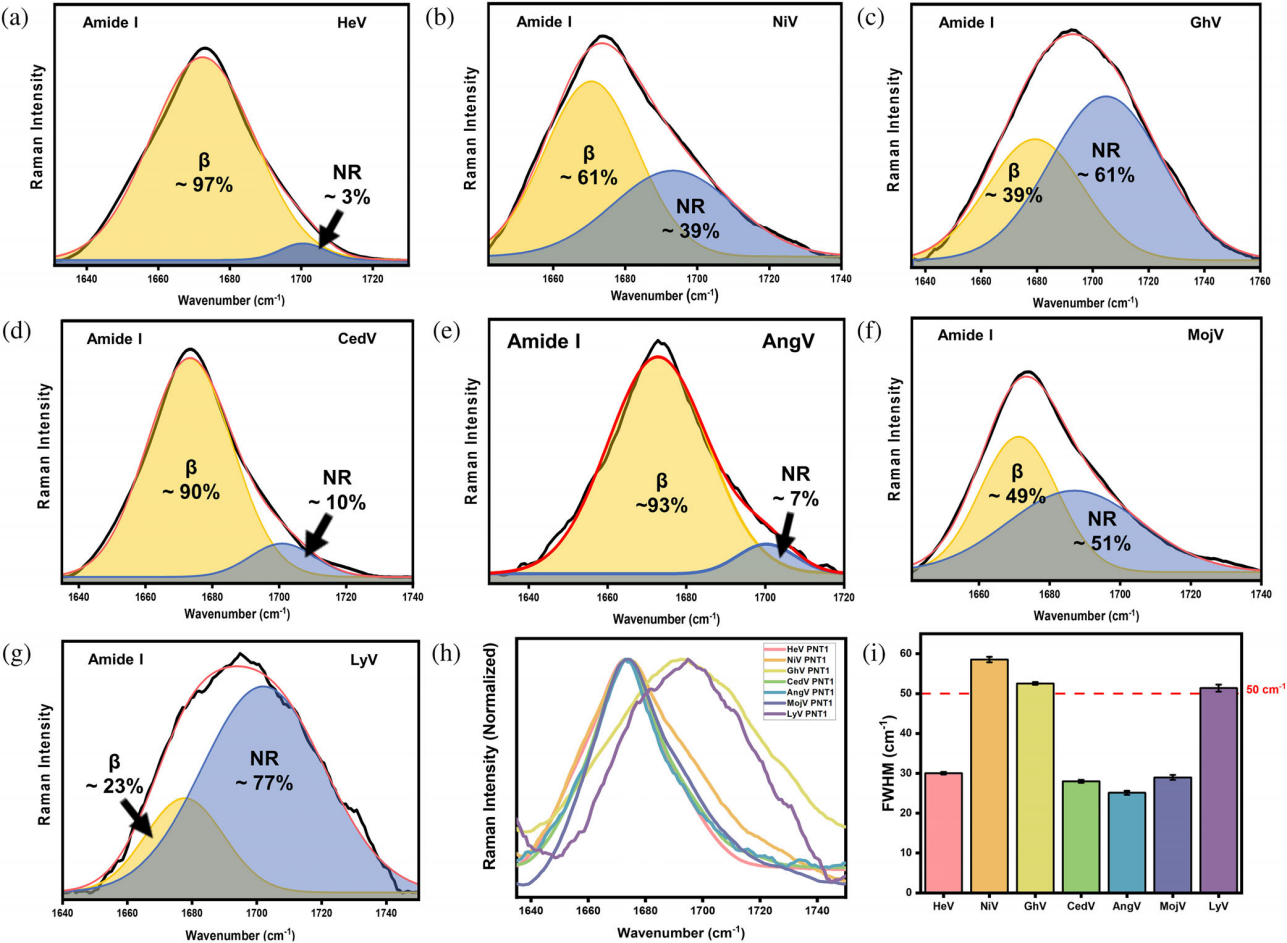
Raman spectroscopic analysis of secondary structure and conformational heterogeneity in PNT1 fibrils. Gaussian deconvolution of the Amide I region (1620–1760 cm^−1^) for PNT1 fibrils from (a) HeV, (b) NiV, (c) GhV, (d) CedV, (e) AngV, (f) MojV, and (g) LyV. Experimental spectra averaged from *n* = 5 accumulations and normalized with respect to Phe (black) and cumulative fits (red) are shown, with individual component contributions corresponding to β‐sheet (yellow) and non‐regular structures (blue), expressed as percentage of area under the curve. (h) Normalized Raman spectra in the Amide I region of all seven PNT1 fibrils, shown in distinct colors. (i) Full width at half maximum (FWHM) values for the Amide I band across samples; increased spectral broadening is associated with greater conformational heterogeneity.

Raman spectral deconvolution of the amide I region across the seven PNT1 homologs revealed distinct variations in β‐sheet and non‐regular structural contributions. Integration of these data with Thioflavin T (ThT) fluorescence, sedimentation assays, and ns‐TEM analyses of mature fibrils provides a comprehensive view of the structural diversity underlying fibril formation among the homologs (Figure 4). While comparing ThT fluorescence and Raman measurements, it is important to note that the two techniques probe different aggregation states. ThT fluorescence was recorded *in situ* and therefore captures contributions from the entire aggregation ensemble, including soluble oligomeric intermediates, whereas Raman spectra were acquired from centrifuged aggregate fractions enriched in insoluble assemblies. HeV PNT1 displays pronounced spectral features consistent with β‐sheet organization and relative structural homogeneity within the amide I region (Figure 6a). However, the ThT signal and sedimentation assays indicate that only limited amounts of mature, insoluble amyloid fibrils are formed (Figure 4a,b). Together, these observations suggest that, although the overall aggregate population is low, the species that do form possess a well‐defined β‐sheet organization. In this context, the lower ThT intensity likely reflects reduced aggregate abundance rather than an absence of β‐structured assemblies. NiV PNT1 displays intermediate β‐sheet contributions accompanied by appreciable non‐regular structural components, indicative of partial conversion toward β‐rich assemblies with increased conformational heterogeneity (Figure 6b). This is consistent with moderate fibril recovery in sedimentation assays and comparatively weak ThT fluorescence, suggesting the presence of labile and partially ordered aggregates. Notably, despite the weak Raman β‐sheet signature and low sedimentable fibril fraction, GhV PNT1 exhibits ThT fluorescence higher than the control, indicating the presence of cross‐β‐containing assemblies, although Raman amide I deconvolution revealed a lower β‐sheet contribution relative to non‐regular structural features (Figure 6c), consistent with structurally heterogeneous or weakly ordered aggregates. CedV PNT1 exhibits one of the most pronounced β‐sheet signatures, characterized by a dominant low‐frequency amide I peak (~1673 cm^−1^) and a relatively narrow bandwidth, indicative of a structurally homogeneous fibril population with a well‐defined hydrogen‐bonded β‐sheet packing (Figure 6d). This observation is consistent with strong ThT fluorescence, a substantial fibrillar fraction in sedimentation assays, and the presence of distinctive fibrils in ns‐TEM (Figure 4a,b,f), collectively supporting the formation of mature cross‐β amyloid fibrils. AngV PNT1 exhibits an amide I profile comparable to that of HeV PNT1 in terms of the contributions from β‐rich assemblies and non‐regular structural components, but with a noticeably narrower peak, indicating a more homogeneous structural organization. ThT fluorescence, sedimentation assays, and TEM analyses all support the formation of fibrillar assemblies in this case (Figure 6e). MojV PNT1 exhibits a similar mixed structural profile, with detectable β‐sheet content alongside significant non‐regular contributions within the amide I envelope (Figure 6f). These features point to structurally heterogeneous aggregates, further supported by moderate ThT fluorescence (Figure 4a), indicating that aggregation does not predominantly yield highly ordered cross‐β architectures. Similarly, LyV PNT1 shows a reduced β‐sheet contribution, suggesting limited backbone rearrangement into extended β‐structures (Figure 6g). LyV PNT1 exhibits an amide I profile comparable to that of GhV PNT1, indicating broadly similar secondary structural features. However, in contrast to LyV PNT1, GhV PNT1 shows reduced ThT fluorescence despite a relatively high sedimentable fibril fraction (Figure 4a,b). This suggests that, although GhV PNT1 forms aggregates of sufficient size to sediment, these assemblies possess reduced accessibility or a lower density of ThT‐binding β‐sheet motifs or grooves. Across several homologs, the amide I band of GhV and LyV exhibits a significant blue shift (Figure 6h). Further analysis of amide I bandwidths revealed distinct differences in the structural heterogeneity of the PNT1 subdomains. HeV, CedV, AngV, and MojV PNT1 exhibit relatively narrow amide I bands (HeV: ~29 cm^−1^ at 1676 cm^−1^; CedV: ~28 cm^−1^ at 1674 cm^−1^; AngV: ~25 cm^−1^ at 1673 cm^−1^; MojV: ~29 cm^−1^ at 1676 cm^−1^), indicative of comparatively homogeneous backbone conformations and structurally consistent assemblies. In contrast, NiV, GhV, and LyV display full widths at half maximum (FWHM) exceeding 50 cm^−1^ (NiV: ~59 cm^−1^ at 1672 cm^−1^; GhV: ~53 cm^−1^ at ~1694 cm^−1^; LyV: ~52 cm^−1^ at 1694 cm^−1^), indicative of substantial conformational heterogeneity and overlapping structural contributions (Figure 6i). Such shifting and broadening is consistent with structurally diverse and metastable assemblies rather than highly ordered β‐sheet‐rich fibrillar species. Meanwhile, a blue shift in the amide I bands of GhV and LyV PNT1 was also observed, consistent with polymorphic or partially ordered assemblies exhibiting heterogeneous backbone organization.

Starting from highly disordered and expanded conformations, MD simulations revealed that all PNT1 proteins rapidly undergo global chain collapse, which persisted beyond 500 ns and converged to a similar reduced (*R*_*g*_) plateau (Figure S3a). Notably, the experimentally determined Raman populations are not expected to quantitatively match the simulated β‐content, as the two approaches probe distinct stages of the aggregation landscape. Specifically, MD simulations capture early intramolecular conformational rearrangements occurring on nanosecond timescales, whereas Raman spectroscopy reflects the structural ensemble formed following intermolecular assembly and subsequent conformational conversion. Notably, HeV PNT1 exhibits a delayed chain collapse accompanied by increased variability between replicates, indicative of a more heterogeneous and fuzzier conformational ensemble. To some extent, CedV and MojV display a comparable plasticity in the first half of the simulation. The compaction observed across all PNT1 constructs correlates with the progressive desolvation of the protein chains, as evidenced by an exponential decrease in solvent accessible surface area (SASA), irrespective of viral origin (Figure S3b). Upon relaxation, viral PNT1 subdomains preferentially adopt compact and hydrophobic conformers that minimize the contacts with surrounding water molecules. While some percentage differences can be observed between the PNT1 subdomains, overall, they all present an increased proportion of β‐sheets and β‐bridges compared to their initial conformational state, which can be associated with a prone‐to‐aggregate behavior (Figure S3c). Furthermore, all variants display significant variability in the formation of β‐sheets, which seemingly indicates that the structural rearrangement they undergo after chain collapse is more of a dynamic process than a steady enrichment in secondary structures (Figure S3c). Such a claim is notably substantiated by the time evolution of the β‐character content for all PNT1 subdomains, showing significant fluctuations over the 1 μs span (Figure S3d). The difference in timescales is critical because aggregation is governed not only by the extent of β‐structure but also by the kinetics with which transient β‐prone states are converted into productive oligomeric and fibrillar species.

Collectively, the amide I analysis indicates that the ability of PNT1 homologs to form ordered β‐sheet‐rich amyloid structures varies considerably despite their shared sequence ancestry.

### 2.5 Amide III analysis complements amide I in capturing structural heterogeneity of PNT1 fibrils

The amide III region (1200–1350 cm^−1^), primarily arising from N–H bending and C–N stretching vibrations, provides a complementary probe of backbone conformation. In contrast to the amide I band, this region is particularly sensitive to backbone torsional angles (φ, ψ) and therefore enables detection of subtle differences in β‐sheet geometry and conformational heterogeneity (Bhattacharya et al., 2013; Lippert et al., 1976; Tuma, 2005). Across the PNT1 homologs, amide III analysis corroborated the structural trends observed in the amide I region while further resolving differences in conformational variability.

**FIGURE 7:**
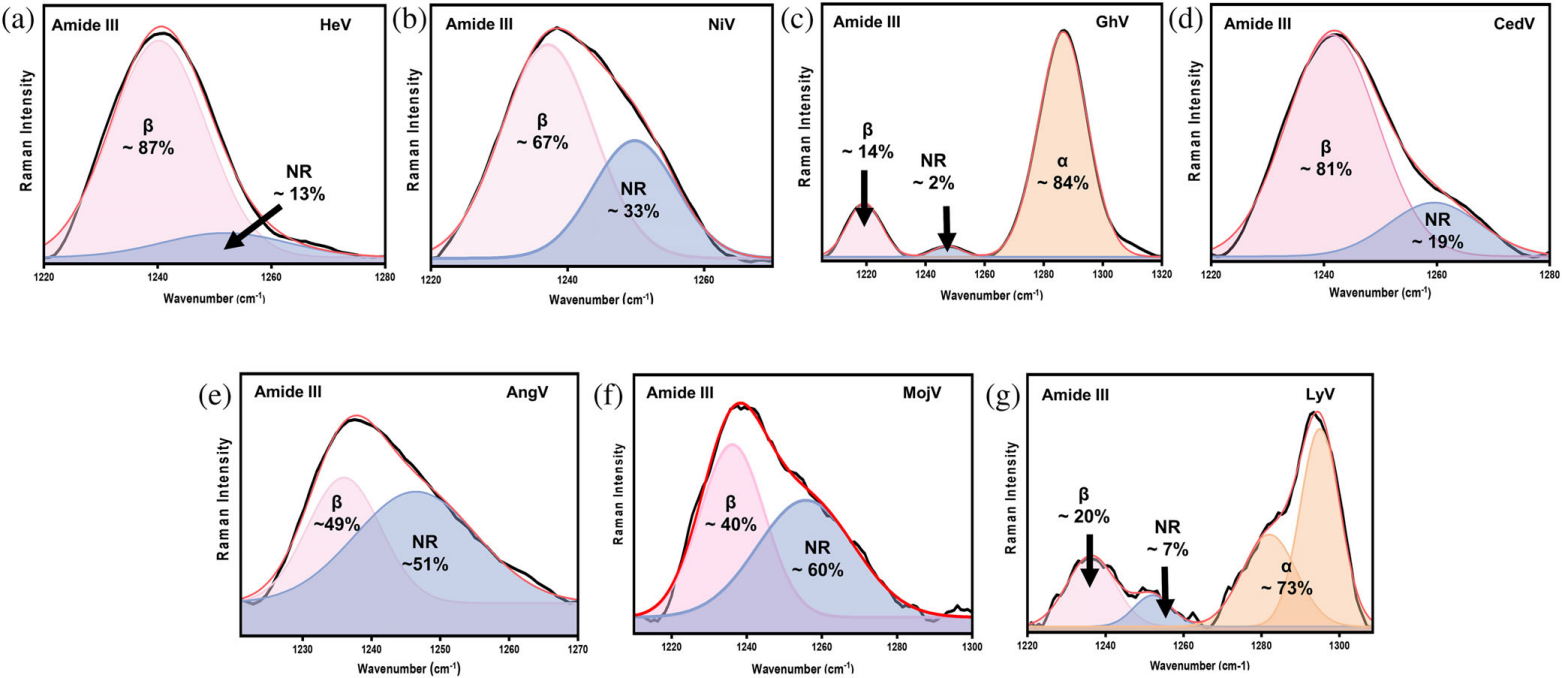
Raman deconvolution of the amide III region reveals secondary structure contributions in PNT1 fibrils. Spectral deconvolution of the Amide III region (1200–1350 cm^−1^) for PNT1 fibrils from (a) HeV, (b) NiV, (c) GhV, (d) CedV, (e) AngV, (f) MojV, and (g) LyV. Experimental spectra averaged from *n* = 5 accumulations and normalized with respect to Phe (black) and cumulative fits (red) are shown, with individual component contributions corresponding to β‐sheet (light pink) and non‐regular structures (light indigo), expressed as percentage of area under the curve.

HeV PNT1 shows an amide III profile consistent with a predominance of β‐sheet structure (Figure 7a). However, when considered alongside other measurements, this likely reflects locally ordered motifs within an overall less mature or weakly aggregated state. NiV PNT1 displays broader amide III bands with overlapping contributions from β‐sheet and disordered structures (Figure 7b), indicative of structurally heterogeneous ensembles, potentially comprising protofibrillar or partially ordered aggregates. The most distinct spectral features were observed for GhV PNT1 (Figure 7c), indicating substantial conformational heterogeneity and consistent with the amide III deconvolution, which revealed reduced β‐sheet contributions alongside notable α‐helical character and minimal non‐regular content (Figure 7c). CedV PNT1 exhibits a well‐defined amide III profile consistent with a dominant β‐sheet conformation, characterized by relatively narrow spectral features (Figure 7d) supporting the presence of a highly ordered fibrillar architecture. These observations further reinforce CedV PNT1 as the most structurally ordered amyloid‐forming variant among the homologs. AngV and MojV PNT1 exhibit significant contributions from non‐regular backbone conformations within the amide III region, consistent with partially structured assemblies (Figure 7e,f). Although β‐sheet features are present, they do not dominate the structural landscape to the same extent as in CedV PNT1. Finally, LyV PNT1 displays an amide III profile comparable to that observed for GhV (Figure 7g). The amide III region additionally revealed contributions from α‐helical structures, consistent with the conformational heterogeneity observed in GhV as well. Together, these data suggest that GhV and LyV PNT1 form structurally diverse or polymorphic assemblies with significant variation in backbone organization.

Backbone torsional angles govern the orientation of peptide donors and acceptors, thereby modulating the formation and geometry of inter‐strand hydrogen bonds. In good agreement with this relationship, MD simulations showed rapid conformational collapse of the monomeric chains on the nanosecond timescale, accompanied by the establishment of transient backbone hydrogen‐bonding networks that shape local structural organization. Although these simulations describe conformational dynamics of monomeric species, the intrinsic backbone propensities observed are expected to influence higher‐order assembly through their effects on backbone packing and intermolecular hydrogen‐bond formation within fibrillar architectures.

**FIGURE 8:**
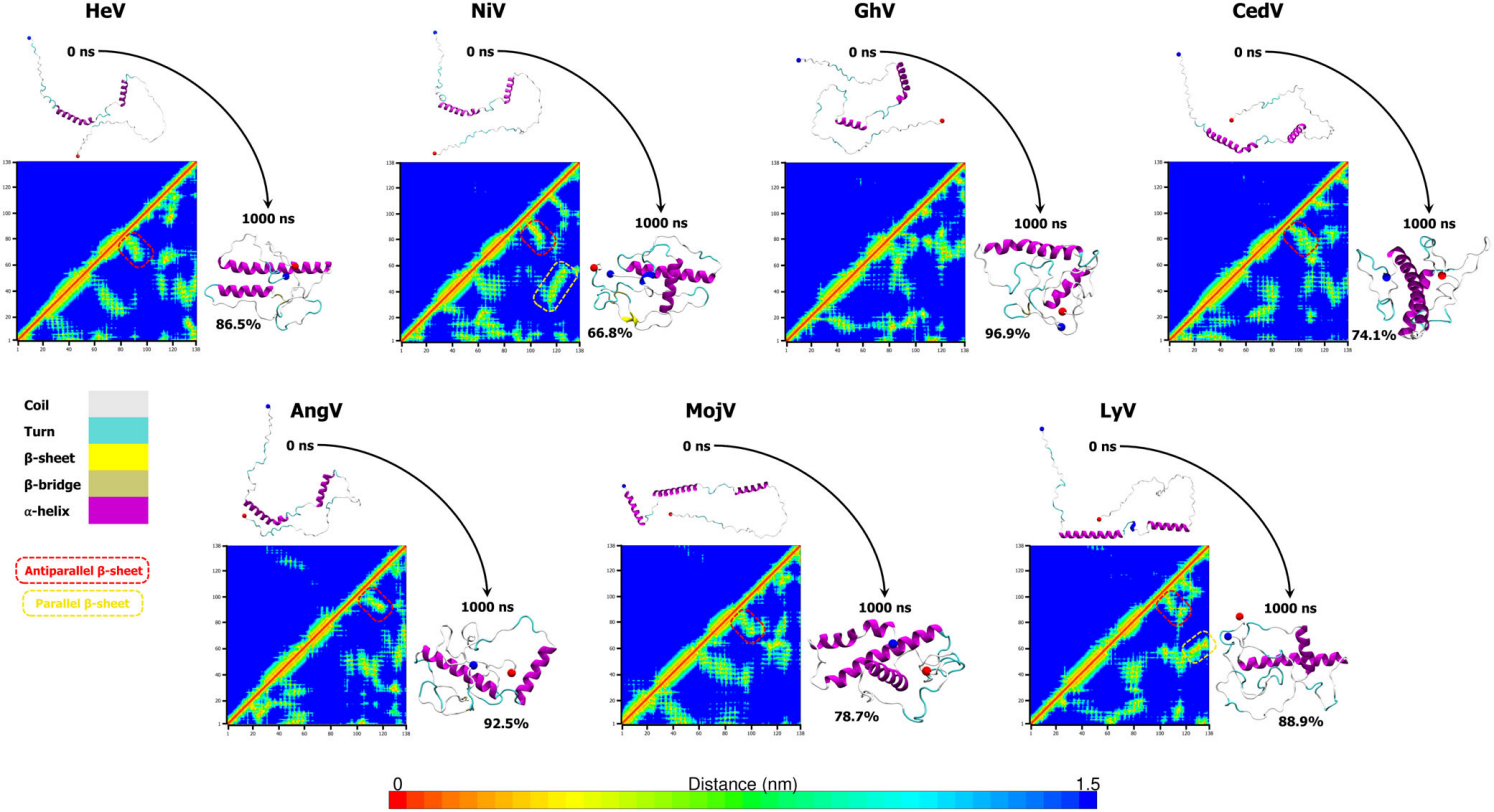
Contact map analysis and dominant conformational states of PNT1 subdomains across *Henipavirus* homologs. Minimum distance residue pairs contact maps of the seven PNT1 subdomains simulated for 1 μs. For each viral PNT1, the contact map of the starting conformation (upper half) is compared to that of the most populated cluster after simulation (bottom half). Antiparallel and parallel β‐sheet features are highlighted on the contact maps by red and yellow dashed frames, respectively. Next to the corresponding contact maps, representative snapshots of the initial state and the central structure of the most populated cluster are represented in cartoons with the N‐ and C‐termini, respectively, shown as blue and red spheres. The different secondary structure elements are colored according to STRIDE assignment: Coil (light gray), turn (teal), extended β‐sheet (yellow), isolated β‐bridge (gold), and α‐helix (magenta). Percentages indicate the population size of the first cluster over the selected trajectories.

Analysis of the MD‐derived backbone hydrogen bond distributions (Figure S4) as anticipated, revealed distinct conformational behaviors across the PNT1 proteins. HeV PNT1 displays a broader hydrogen bond distribution (Figure S4), consistent with dynamic and metastable assemblies. Although Raman signatures suggest β‐sheet features (Figures 6a and 7a), the heterogeneity in hydrogen bonding suggests that these interactions remain transient and fail to consolidate into fully mature fibrillar architectures. NiV, GhV, CedV, and AngV PNT1 show narrower and stable backbone hydrogen bond distributions, suggesting the formation of homogeneous and structurally stable backbone conformations within their assembled states, despite differences in the absolute number of hydrogen bonds. In contrast, MojV PNT1 displayed a distribution shifted toward a population of higher hydrogen bond content while maintaining a relatively narrower and stable profile. Such discrepancy noticeably originates from persistent intramolecular hydrogen‐bonding interactions stabilizing the additional α‐helical segment present in the starting model (see section 7 and Figure 8). LyV PNT1 displays structural heterogeneity with a broader hydrogen bond distribution and increased spread, likewise indicative of substantial conformational diversity and less persistent backbone hydrogen bonding, consistent with polymorphic or structurally diverse aggregates (Figure S4).

In addition to backbone structural features, Raman spectroscopy provides insight into side‐chain environments and molecular packing through analysis of aromatic residues and aliphatic deformation modes. To probe local environments of tyrosine residues, the intensity ratio of the Tyr Fermi doublet (I_850_/I_830_) was examined (Figure S5). This doublet arises from Fermi resonance between the ring‐breathing vibration and the overtone of an out‐of‐plane ring‐bending mode of the phenolic ring and is sensitive to hydrogen bonding and solvent exposure (Hernández et al., 2016). HeV, NiV, GhV, AngV, and CedV PNT1 display lower tyrosine doublet ratios, indicative of reduced solvent accessibility and burial of aromatic side chains within more tightly packed assemblies (Figure S5). In contrast, MojV and LyV PNT1 exhibit an elevated tyrosine doublet ratio (I_850_/I_830_ ≥2), consistent with increased solvent exposure of Tyr residues and a more hydrated local environment. Interestingly, sequence comparison reveals that MojV and LyV PNT1 uniquely contain an additional Tyr residue within the region preceding the CAR (Figure S6a). While the elevated I_850_/I_830_ ratios cannot be explained solely by the presence of a single additional Tyr residue, this shared sequence feature points to differences in aromatic residue patterning that may contribute to the increased solvent accessibility of Tyr side chains. These observations suggest that residue distribution, rather than Tyr content alone, influences the local hydration environment within henipavirus PNT1 assemblies.

Tryptophan Raman bands provide complementary information on local microenvironments (Hernández et al., 2024; Miura et al., 1988). The band near ~880 cm^−1^, associated with indole N–H bending (Table S1), is sensitive to hydrogen bonding interactions with the surrounding solvent (Hernández et al., 2024). CedV, LyV, AngV, and GhV PNT1 exhibit features consistent with hydrogen‐bonded indole groups, suggesting partial solvent exposure or involvement in polar interactions. In contrast, NiV, HeV, and MojV PNT1 show a blue shift of this band toward ~890 cm^−1^, indicative of reduced hydrogen bonding and increased burial of tryptophan residues within hydrophobic cores. These observations reveal distinct local microenvironments across PNT1 assemblies, with GhV, CedV, AngV, and LyV retaining more solvent‐exposed indole groups, whereas HeV, NiV, and MojV displayed spectral shifts consistent with increased burial within the hydrophobic cores. Notably, the Trp residues are conserved in both number and approximate sequence position across all seven PNT1 proteins (Figure S6a). The observed spectral differences therefore cannot be attributed to changes in Trp content but instead reflect differences in the local environments experienced by these conserved residues within the assembled state. These findings indicate that sequence‐dependent variations elsewhere in the PNT1 domain give rise to distinct packing arrangements and hydration environments, resulting in differential solvent accessibility of otherwise conserved indole groups. The 1440–1470 cm^−1^ spectral region provides a sensitive probe of residue‐specific packing within PNT1 fibrils and can be directly interpreted in light of the conformational collapse, desolvation, and hydrogen bond reorganization observed in the simulations. The 1440–1470 cm^−1^ region provides a concise, residue‐specific signature of fibril organization for four PNT1 subdomains (Figure S7a–d). HeV, NiV, CedV, and AngV PNT1s display a dominant band at ~1447 cm^−1^ (δ(NH), guanidinium) and a shoulder at ~1465 cm^−1^ (CH_2_/CH₃ deformation), whose relative contributions report on the balance between arginine‐mediated electrostatic networks and hydrophobic side‐chain packing (Bhunia et al., 2016; Garrido et al., 2013). HeV (with a ~53% contribution of the δ(NH) guanidinium moiety) displays balanced contributions, indicating mixed architectures where electrostatic and hydrophobic interactions coexist, consistent with intermediate compaction and variable β‐structure content (Figure S7a). The strengthened ~1447 cm^−1^ band across all these subdomains reflects the central role of guanidinium groups in increased side‐chain packing and reduced solvent exposure within the aggregates, potentially reflecting stabilization by intermolecular hydrogen‐bonding and salt‐bridge interactions. NiV and CedV (with a ∼63% contribution of the δ(NH) guanidinium moiety) (Figure S7b,c) also exhibit arginine‐driven architectures; NiV combines this with buried tryptophan (∼890 cm^−1^), whereas CedV retains partial solvent exposure. In contrast, AngV (with a ∼34% contribution of the δ(NH) guanidinium moiety) (Figure S7d) is dominated by CH_2_/CH₃ modes, reflecting hydrophobic packing with more solvent‐exposed arginine and tryptophan residues; AngV further suggests a distinct aliphatic packing geometry. Interestingly, sequence comparison indicates that AngV contains an Arg residue in the region preceding the CAR, distinguishing it from the other PNT1 subdomains analyzed in this study (Figure S6a). Although this sequence feature may influence the local interaction landscape, the reduced guanidinium contribution in AngV suggests that this Arg residue does not simply increase buried arginine packing but may instead alter the spatial organization of electrostatic contacts or shift the fibril toward a distinct packing register. Reliable deconvolution of this region could not be performed for GhV, MojV, and LyV because of reduced signal‐to‐noise in the 1440–1470 cm^−1^ window; these spectra were therefore excluded from quantitative comparison to avoid overinterpretation.

### 2.6 Sequence evolution uncouples phylogenetic relatedness from aggregation behavior

Analysis of Tyr, Trp, and Arg residue distribution (Figure S6a), of phylogenetic relationships (Figure S6b), and of the integrated biophysical dataset gathered in this study reveals that evolutionary relatedness is only weakly reflected in the aggregation landscape of PNT1. Although all seven homologs retain the conserved CAR, indicating inheritance of a common fibrillogenic nucleus, the mechanisms governing self‐assembly diverge substantially across the genus.

Within the *Henipavirus* clade—comprising HeV, NiV, GhV, CedV, and AngV (Figure S6b)—aggregation behavior varies remarkably despite relatively close evolutionary relationships. HeV rapidly forms metastable higher‐order oligomers that subsequently decay, whereas the closely related NiV follows a controlled equilibrium‐driven pathway characterized by stable oligomer populations and gradual monomer recruitment. CedV, despite clustering within the same evolutionary branch, exhibits the most efficient amyloidogenesis, producing highly ordered cross‐β fibrils with the strongest ThT fluorescence and highest insoluble fibril fraction. GhV instead preferentially generates heterogeneous protofibrillar intermediates, while AngV displays a markedly delayed nucleation process followed by the formation of comparatively homogeneous mature fibrils. Thus, even among closely related henipaviruses, aggregation kinetics, intermediate stability, and fibril architecture are highly divergent.

A similar trend is observed for the more distantly related LyV and MojV, which cluster separately from the canonical henipaviruses (Figure S6b). Despite their close phylogenetic relationship, these two homologs do not converge on a common aggregation mechanism. MojV primarily forms persistent transient oligomers with limited growth, whereas LyV rapidly generates heterogeneous higher‐order assemblies characterized by broad Raman signatures and polymorphic fibrils. Therefore, neither close nor distant evolutionary relationships consistently predict aggregation behavior.

Instead, the experimental observations correlate better with the residue organization shown in Figure S6a than with phylogenetic clustering. The CAR and the positions of the tryptophan residues are highly conserved across all homologs, explaining the universal ability of the proteins to undergo fibrillation. In contrast, substantial variation is observed in the spatial distribution of Arg and Tyr residues surrounding the CAR. These sequence differences coincide with pronounced changes in conformational heterogeneity observed by MD simulations, which subsequently manifest as distinct oligomerization pathways detected by TDA and culminate in different β‐sheet organization, side‐chain packing, and aromatic residue environments in the mature fibrils revealed by Raman spectroscopy. Conserved Trp residues also experience markedly different local environments despite occupying equivalent sequence positions, further indicating that the surrounding sequence context, rather than residue conservation itself, governs the structural outcome of aggregation.

Collectively, these findings indicate that viral evolution has conserved the amyloidogenic nucleus while diversifying the molecular grammar encoded by the surrounding intrinsically disordered sequence. Rather than following phylogenetic relationships, the aggregation landscape is dictated by sequence‐dependent residue patterning that reshapes the conformational ensemble of the monomer, modulates intermolecular interaction networks, and ultimately determines pathway selection and fibril architecture. These results therefore establish that local sequence context, rather than evolutionary distance, is the principal determinant of PNT1 self‐assembly within the henipavirus genus.

### 2.7 Intramolecular contact mapping of relaxed PNT1 proteins

For all PNT1 subdomains, MD trajectories were collected starting from an initial and unrelaxed AlphaFold2 (AF2) model that, in all cases, was found to encompass an N‐terminal kinked α‐helix, reflecting existing structural information on the NiV RNA‐free nucleoprotein‐phosphoprotein complex (PDB code 4CO6) (Ker et al., 2021). Examination of the simulation snapshots extracted from the most populated clusters (Figure 8) revealed that the modeled α‐helices remained mostly folded upon relaxation and were found in closer proximity within the collapsed structures of the PNT1 proteins, although all initial helical kinks were generally lost over time. In addition, the sporadic appearance of turns and β‐elements highlights the progressive folding of previously disordered regions. This structural ordering was further supported by the corresponding residue‐pair contact maps (Figure 8). Indeed, long‐range interactions, absent after energy minimization and system equilibration, emerge in the relaxed states of the PNT1 proteins together with new short‐range contacts. These observations indicate that the protein chains tend to maximize multiple and multivalent intramolecular interfaces, thereby remodeling their conformational landscape and promoting folding. Such intramolecular networking involved not only the CAR motif but also the C‐terminal flanking region. Notably, structural features characteristic of antiparallel (red dashed frame) and parallel (yellow dashed frame) β‐sheets became particularly evident within the latter. Overall, these data corroborate the previous findings: *Henipavirus* PNT1 subdomains undergo structural chain collapse and desolvation, accompanied by an increase in both short‐ and long‐range intramolecular contacts, notably through the formation of new secondary structures, such as β‐sheets and β‐bridges, leading to the generation of aggregation‐prone intermediates. Nevertheless, local discrepancies and construct‐specific features, also evident in the contact maps, allow discrimination among PNT1 variants, reflecting the experimentally observed diversity in fibrillar core organization, architecture, and morphology.

## 3 CONCLUSIONS

As summarized in Table 1, combined experimental and computational analyses revealed that *Henipavirus* PNT1 subdomains, despite harboring a conserved CAR, exhibit markedly divergent aggregation behaviors and fibril architectures. By integrating atomistic MD simulations with TDA and Raman spectroscopy, we demonstrate that the presence of a shared fibrillogenic motif does not dictate a uniform assembly pathway. Instead, fibrillation is strongly modulated by the surrounding sequence context, particularly the intrinsically disordered regions flanking the CAR, which display substantial conformational flexibility during the simulations, while the CAR itself remains comparatively rigid across viruses. These sequence‐dependent differences are reflected experimentally in the early stages of aggregation, where TDA resolves distinct timelines of oligomer formation, differential monomer recruitment, and variations in the growth of higher‐order species under identical solution conditions. Raman spectroscopy further reveals that these divergent assembly pathways culminate in structurally distinct end states, ranging from highly ordered cross‐β fibrils to more heterogeneous and partially disordered aggregates, accompanied by differences in β‐sheet organization, hydration, aromatic residue exposure, and side‐chain packing. Noteworthy, the observed differences in aggregation pathways and fibril organization cannot be ascribed to phylogenetic relationships among the *Henipavirus* members.

**TABLE 1:** Integrated summary of the aggregation behavior of the seven Henipavirus PNT1 homologs.

PNT1 homologs	TDA (early assembly)	MD (conformational landscape)	Raman (mature fibrils)	ThT/TEM	Overall interpretation
HeV	Rapid formation of transient large oligomers	Highest heterogeneity; delayed collapse; broad Rg/H‐bond distributions	Ordered β‐sheets; narrow amide I; buried Trp	Low ThT; few fibrils; sparse fibrils observed by ns‐TEM	Metastable pathway with inefficient fibril maturation
NiV	Stable equilibrium oligomers	Compact ensemble; low heterogeneity	Intermediate β‐sheet; broad amide I; buried Trp	Moderate ThT; few fibrils	Controlled nucleation‐growth pathway
GhV	Early protofibrillar species	Moderate heterogeneity	Broad, blue‐shifted amide I; reduced β‐sheet	Moderate ThT; high fibril fraction; moderate fibrillar networks	Rapid formation of polymorphic fibrils
CedV	Progressive oligomer growth	Compact ensemble; increased β‐content	Strongest β‐sheet signature; narrow amide I	Highest ThT; abundant fibrils	Most efficient and ordered amyloid formation
AngV	Delayed nucleation	Restricted conformational landscape	Ordered β‐sheet; narrow amide I	Moderate‐high ThT; sparse fibrillar assemblies	Slow nucleation followed by homogeneous fibrils
MojV	Stable transient oligomers	Expanded conformations; persistent α‐helix	Mixed β‐sheet/disordered structure	Moderate ThT; moderate abundance of mature fibrils	Alternative pathway with transient intermediates
LyV	Early protofibrillar species	Moderate heterogeneity	Broad, blue‐shifted amide I; reduced β‐sheet	Moderate ThT; abundant fibrillar assemblies	Rapid formation of heterogeneous fibrils

Importantly, the observed fibril structural heterogeneity cannot be explained solely by differences in the abundance of Tyr, Trp, and Arg residues. Indeed, sequence analysis indicates that variations in the spatial distribution of Tyr, Trp, and Arg residues give rise to distinct interaction networks involving π–π, cation–π, hydrogen bonding, and electrostatic contacts. Conserved Trp residues experience markedly different local environments across fibrils, while differences in Tyr accessibility and guanidinium‐associated Raman signatures correlate with residue patterning rather than absolute residue content. These findings support a model in which sequence‐encoded interaction landscapes outside the conserved amyloidogenic core govern conformational sampling in the monomeric ensemble and subsequently direct the assembly pathway toward distinct fibrillar states. Viral evolution has therefore diversified the aggregation landscape of the V and W proteins not by altering the conserved CAR itself but by modulating the molecular grammar encoded in the surrounding disordered sequence. Such variability in aggregation behavior may have important consequences for viral replication, host–pathogen interactions, and pathogenicity. More broadly, this work highlights how subtle changes in residue patterning can reshape the energy landscape of IDPs and shows that conserved amyloidogenic motifs are insufficient predictors of assembly behavior in the absence of their sequence context. Elucidating these sequence‐encoded determinants of viral protein fibrillation provides a framework for understanding disorder‐driven self‐assembly in viral systems and may inform future strategies targeting aggregation‐mediated mechanisms in viral disease.

## 4 MATERIALS AND METHODS

### 4.1 Protein expression and purification

The recombinant plasmids encoding the seven *Henipavirus* PNT1 subdomains with an N‐terminal hexahistidine tag have already been described (Gondelaud, Bignon, et al., 2025). Plasmids were transformed into *Escherichia coli* T7pRos cells. The PNT1 constructs were expressed and purified as described previously. Briefly, proteins were purified under denaturing conditions (6 M urea) using Sepharose resin loaded with NiSO_4_ affinity chromatography (Cytiva), followed by anion‐exchange chromatography on a diethylaminoethyl (DEAE) column (Cytiva) and size‐exclusion chromatography (SEC).

Expression cultures were seeded from 100 mL overnight precultures grown in lysogeny broth (LB) supplemented with 100 μg/mL ampicillin and 34 μg/mL chloramphenicol at 37°C and 200 rpm. Precultures were used to inoculate 2 L of terrific broth (TB) (1:40 dilution), and cultures were grown to an OD₆₀₀ of 0.6–0.8 prior to induction with 1 mM isopropyl β‐D‐1‐thiogalactopyranoside (IPTG) for 4 h at 37°C. Cells were harvested from 2 L culture by centrifugation (6000×*g*, 10 min, 4°C) and resuspended in 30 mL of Buffer A (20 mM HEPES, 150 mM NaCl, 6 M urea, pH 7.2).

Cell lysis was performed by sonication using a Sonics Vibra‐Cell sonicator (Sonics & Materials, VCX‐500UK‐230) equipped with a 3 mm microtip. Samples were sonicated at 40% amplitude using 30 s ON/30 s OFF pulses for a total cycle time of 2 min. This was followed by centrifugation (15,000×*g*, 30 min, 4°C). The supernatant was incubated with 5 mL of pre‐equilibrated Ni–NTA resin (Cytiva) with Buffer A under gentle agitation (15 rpm) for 45 min at 4°C. The resin was first washed with 25 column volumes (CVs) of Buffer B (20 mM HEPES, 1 M NaCl, 6 M urea, pH 7.2), then with 25 CVs of Buffer C (20 mM HEPES, 50 mM NaCl, 6 M urea, pH 7.2). Proteins were eluted with Buffer C containing 0.25 M imidazole.

Eluted proteins were further purified by DEAE anion‐exchange chromatography (Cytiva). The column was pre‐equilibrated in Buffer C, and proteins were eluted using a salt gradient using 20 mM HEPES, 500 mM NaCl, 6 M urea, pH 7.2. The proteins were further purified by size‐exclusion chromatography (SEC) on a HiLoad Superdex 75 16/600 column (Cytiva) equilibrated in Buffer A. Fractions were pooled, concentrated using 3 kDa cutoff filters (Merck Millipore), and stored at −20°C in 6 M urea. Protein concentrations were determined spectrophotometrically by measuring the absorbance at 280 nm and using the extinction coefficients as calculated via the ProtParam program from the EXPASY server.

### 4.2 Taylor dispersion analysis (TDA)

Taylor dispersion analyses (TDA) of the seven PNT1 subdomains were performed as previously described (Deleanu et al., 2021) using an Agilent 7100 capillary electrophoresis (CE) system (Agilent Technologies, Waldbronn, Germany) equipped with a diode array UV detector and a Zetalif LED‐induced fluorescence detector (LEDIF) from Adelis (Toulouse, France). Experiments were conducted in bare fused‐silica capillaries (Polymicro Technologies, USA) with a total length of 60 cm, an internal diameter of 49.6 μm, and a fluorescence detection window located 39 cm from the inlet.

Prior to use, capillaries were conditioned by flushing with 1 M NaOH for 5 min followed by ultrapure water for 10 min. Between analyses, the capillary was flushed with 1 M NaOH for 150 s and ultrapure water for 300 s. Capillaries were then pre‐saturated by flushing with a 20‐fold diluted sample for 30 s, followed by a 2 min rinse with Buffer D (20 mM HEPES, 150 mM NaCl, pH 7.2) (Roca et al., 2024).

Fluorescence detection was performed at a 275 nm excitation wavelength, and the emission light was collected using a high‐pass filter between 300 and 450 nm. Since all seven proteins possess tryptophan, tyrosine, and phenylalanine residues in their sequence, the latter detector could be used for native fluorescence and the detection of the proteins. Samples were hydrodynamically introduced by pressure injection from the inlet end of the capillary (30 mbar for 6 s), corresponding to an injected volume of approximately 5.6 nL (<1% of the capillary volume to the detection window). The mobile phase consisted of Buffer D (viscosity at 37°C ~8.0 × 10^−4^ Pa·s). Samples were mobilized in the capillary by applying a pressure of 50 mbar; the temperature was maintained at 37°C. The vial carousel was thermostatted at 37°C using a Julabo CORIO CD‐600F, an external circulating water bath.

Protein samples were prepared by rapid dilution from a 600 μM stock to a final concentration of 50 μM protein and 0.5 M urea in Buffer D and in a final volume of 1 mL. Samples were incubated at 37°C in the CE autosampler, and aggregation was monitored by injecting aliquots three times every 85 min. Taylorgrams were acquired using Agilent ChemStation software and processed using Microsoft Excel. When required, elution peaks were fitted to a sum of up to two Gaussian functions. Taylorgrams were analyzed using first‐order exponential fits, and when required, peak profiles were deconvoluted using Gaussian functions (Deleanu et al., 2023).

### 4.3 Raman spectroscopy

Raman spectra were acquired using an inVia Raman microscope (Renishaw, UK). Samples were prepared under the same conditions used for TDA experiments. Aggregation reactions were conducted in 10 × 10 mm quartz cuvettes and incubated at 37°C in a thermostated water bath. 50 μM protein samples were prepared by diluting a 600 μM stock into Buffer D. Samples were incubated for more than 100 h to allow aggregation and the formation of higher‐order mature fibrils.

Aggregated samples were centrifuged at 16,400×*g* for 30 min at room temperature, and the supernatant was carefully removed. Pellets were washed twice with 200 μL of Milli‐Q water, with centrifugation at 16,400×*g* for 30 min between washes. Pellets were air‐dried overnight, resuspended in 10 μL Milli‐Q water, and drop‐cast onto aluminum foil–covered glass slides. After drying, spectra were collected using a 50× objective lens (Nikon, Japan) and excitation with a 785 nm near‐infrared laser (10 s exposure time, 5% laser power, 25 mW). Experiments were repeated with three different batches of protein samples subjected to aggregation for 100 h. Data were acquired using the Wire 3.4 software provided with the Raman spectrometer. The collected Raman spectra were baseline corrected using the cubic spline interpolation method and smoothened using Wire 3.4 software and plotted using Origin.

### 4.4 Thioflavin T (ThT) fluorescence assays

Those fluorescence measurements were performed on the aggregated protein samples, as obtained under the same conditions used for TDA and Raman studies (i.e., on samples at 50 μM in Buffer D *plus* 0.5 M urea incubated for 100 h at 37°C). A PHERAstar FSX plate reader (BMG Labtech) and black 96‐well plates with transparent flat bottoms (Greiner, 655,096) were used. The excitation wavelength was set at 440 nm, and the emission was recorded at 480 nm. ThT was at 50 μM. Experiments were conducted in triplicate across three independent reactions of each viral PNT1 sample, and data are reported as mean ± standard deviation.

### 4.5 Sedimentation assays

Protein aggregation was assessed by sedimentation assays to separate soluble species from insoluble fibrils. Samples were centrifuged at high speed (16,400×*g*, 30 min, 25°C). The supernatant (soluble fraction) was carefully collected, and the pellet (insoluble fibrillar fraction) was resuspended in an equal volume of buffer. Protein concentration in the supernatant was quantified spectrophotometrically by measuring the absorbance at 280 nm. Fraction of soluble species was calculated from the protein concentration remaining in the supernatant relative to the initial total concentration, and fibril percentage was determined from the protein concentration of the sedimented fraction. Data are reported as mean ± s.d. from 9 independent replicates.

### 4.6 Negative‐staining transmission electron microscopy (ns‐TEM)

Aggregated protein samples were diluted in Buffer D to a final protein concentration of 4 μM prior to grid preparation. Carbon‐coated copper grids (300 mesh, 3 mm diameter; TAAB) were first rendered hydrophilic by plasma glow discharge for 20 s using a GloQube system (Quorum Technologies) operated at a current of 25 mA. Immediately after treatment, 3.5 μL of the diluted protein sample was applied to the grid and allowed to adsorb for 1 min. Excess liquid was removed by blotting with filter paper. Grids were subsequently washed three times with Buffer D to remove unbound material. Negative staining was performed by incubating the grids with 35 μL of 1% (w/v) uranyl acetate solution for 1 min. Excess stain was removed by blotting, and grids were allowed to air‐dry overnight at room temperature. Imaging was performed using a TECNAI T12 Spirit transmission electron microscope (Thermo Fisher Scientific) operated at an accelerating voltage of 120 kV and equipped with a Veleta 2 K × 2 K CCD camera (Olympus).

### 4.7 Structure modeling and system preparation prior to simulation

From the corresponding sequence, the starting three‐dimensional structure of each of the seven viral PNT1 proteins was modeled using MMseqs2‐AlphaFold2 (ColabFold) and selected based on the lowest count of intramolecular contact biases among the top‐ranked models (Mirdita et al., 2022). Using the CHARMM‐GUI suite, side‐chain atoms were adequately protonated, and the C‐terminus was capped with an amide group before separately centering the protein chains in cubic water boxes and adding a 10 Å buffer of water to their edges (Jo et al., 2008; Park et al., 2023). To mimic the experimental conditions, urea molecules at a concentration of 0.6 M were enforced into the solvation boxes along with NaCl at 150 mM. According to the protein net charge and the box volume of each system, the required number of ions and urea molecules was added. All parameters are summarized in Table S2.

### 4.8 All‐atom equilibrium molecular dynamics (MD) simulation

Protein atoms and ions were modeled according to the CHARMM36m force field used with the CHARMM‐modified TIP3P solvation model for water molecules (Huang et al., 2017; Hwang et al., 2024). MD steps and simulations were carried out in independent triplicates with GROMACS 2023.1 suite implemented with GPU (CUDA 11.7.0) accelerators (Abraham et al., 2015; Van Der Spoel et al., 2005). By applying hydrogen mass repartition (HMR) on the initial topologies, Newton's equations of motion were allowed to be integrated using an increased timestep of 4 fs (Gao et al., 2021; Hopkins et al., 2015). All the bonds comprising hydrogen atoms were additionally constrained to their proper equilibrium length with the LINCS algorithm (Hess et al., 1997) System minimization was performed for a maximum of 5000 steps with the steepest descent algorithm before thermalization, equilibration, and propagation in the isothermal‐isobaric (NPT) ensemble with the leap‐frog integrator (Müller & Brown, 1979; Van Gunsteren & Berendsen, 1988). In that purpose, pressure of 1 bar and temperature of 310.15 K were respectively enforced using a C‐rescale barostat and modified Berendsen thermostat (V‐rescale) (Bernetti & Bussi, 2020; Bussi et al., 2007). Short‐range non‐bonded interactions were computed with a distance cut‐off of 1.2 nm, whereas Particle Mesh Ewald (PME) summation was applied for long‐range coulombic interactions (Bussi et al., 2007). The triplicated production MD runs were propagated in periodic boundary conditions (PBC) in all three dimensions for a duration of 1 μs each. Atom coordinates and energies were recorded every 100 ps.

Modules directly invoked from the GROMACS suite were conjunctively used with in‐house built Python scripts for trajectory analysis and clustering. For each system, time‐dependent and per‐residue variables and their associated probability density function (PDF) were computed and averaged across the replicates. Root‐mean‐square fluctuation (RMSF) was determined for each amino acid by taking the time‐averaged structure as a reference. Over the replicates, a total of 21,000 frames were clustered by defining a 5.0 Å RMSD cut‐off between the nearest neighbors. Secondary structure classes were defined using the STRIDE algorithm implemented in the Timeline VMD plugin: turns (i–i + 3 turns), extended β‐sheet, isolated β‐bridge (usually i–i+>6), α‐helix (i–i + 4 helix), 3_10_‐helix (i–i + 3 helix), π‐helix (distorted or bulged i–i + 5 helix), and coil. Trajectory visualization and snapshot rendering were achieved with the VMD molecular graphics program (Humphrey et al., 1996).

### 4.9 Bioinformatic analyses

The first 110 amino acid residues of the P/V/W proteins from HeV, NiV, MojV, CedV, GhV, LyV, and AngV were aligned using ClustalW (Thompson et al., 1994) and ESPript (Robert & Gouet, 2014).

## AUTHOR CONTRIBUTIONS

**Sonia Longhi:** Writing – review and editing; supervision; validation; project administration; funding acquisition; conceptualization. **Joseph Chamieh:** Conceptualization; validation; writing – review and editing; supervision. **Harshita Sawdekar:** Writing – original draft; investigation; conceptualization; formal analysis. **Frank Gondelaud:** Writing – review and editing. **Julien Mignon:** Investigation; formal analysis; writing – review and editing. **Anuja Walimbe:** Writing – review and editing. **Samrat Mukhopadhyay:** Funding acquisition; writing – review and editing.

## Supporting information


**FIGURE S1.** SDS‐PAGE (15%) analysis of the seven purified PNT1 subdomains. MM: molecular mass markers, from top to bottom: 250–10 kDa.
**FIGURE S2.** Average Raman spectrum of mature fibrils of seven PNT1 subdomains after 100 h incubation.
**FIGURE S3.** MD simulation‐based estimation of the time evolution of the radius of gyration, solvent‐accessible surface area (SASA), time‐averaged β character, and time evolution of the β character.
**FIGURE S4.** Distribution of backbone H bonds, defined as a probability density function (PDF), for the PNT1 subdomains.
**FIGURE S5.** Intensity ratios of the tyrosine Fermi doublet (I_850_/I_830_), which report on solvent‐mediated hydrogen bonding propensity of the phenolic (–OH) group, for the seven PNT1 subdomains.
**FIGURE S6.** Schematic representation of Tyr, Trp and Arg residues conservation across the seven *Henipavirus* PNT1 subdomains.
**FIGURE S7.** Gaussian deconvolution of the 1420–1490 cm^−1^ region highlights guanidinium and aliphatic side‐chain contributions in PNT1 fibrils.
**TABLE S1.** Peak assignment of some selected modes of Raman spectra of the seven *Henipavirus* PNT1 subdomains.
**TABLE S2.** Summary of the MD metrics, parameters, and durations for the seven simulated PNT1 subdomains.

## Data Availability

The data that support the findings of this study will be publicly available in Dat'AMU at https://entrepot.recherche.data.gouv.fr/dataverse/univ-amu after acceptance of this research article.
