# Supplementary material for: Sequence‐encoded determinants drive distinct early aggregation pathways and different structural outcomes in PNT1 fibrils across Henipavirus members
Source: Protein Sci. 2026 Aug 11;35(9):e70758. doi: 10.1002/pro.70758 (PMC13459193; doi:10.1002/pro.70758)
Supplement: Supplementary file 1 [file PRO-35-e70758-s001.pdf]

Supporting Material

Sequence-encoded determinants drive distinct early aggregation pathways and different structural outcomes in PNT1 fibrils across *Henipavirus* members

Harshita Sawdekar[1,2]* , Julien Mignon[1], Frank Gondelaud[1] , Anuja Walimbe[3], Samrat Mukhopadhyay[3,4,5], Joseph Chamieh[2]*, and Sonia Longhi[1]*

[1] Aix-Marseille Université, CNRS, Architecture et Fonction des Macromolécules Biologiques (AFMB), UMR 7257, Marseille, France;

[2] IBMM— IBMM, University of Montpellier, CNRS, ENSCM, 34095 Montpellier, France;

[3] Centre for Protein Science, Design, and Engineering; [4] Department of Chemical Sciences; and [5] Department of Biological Sciences, Indian Institute of Science Education and Research (IISER), Mohali, Punjab, India.

***Corresponding authors**

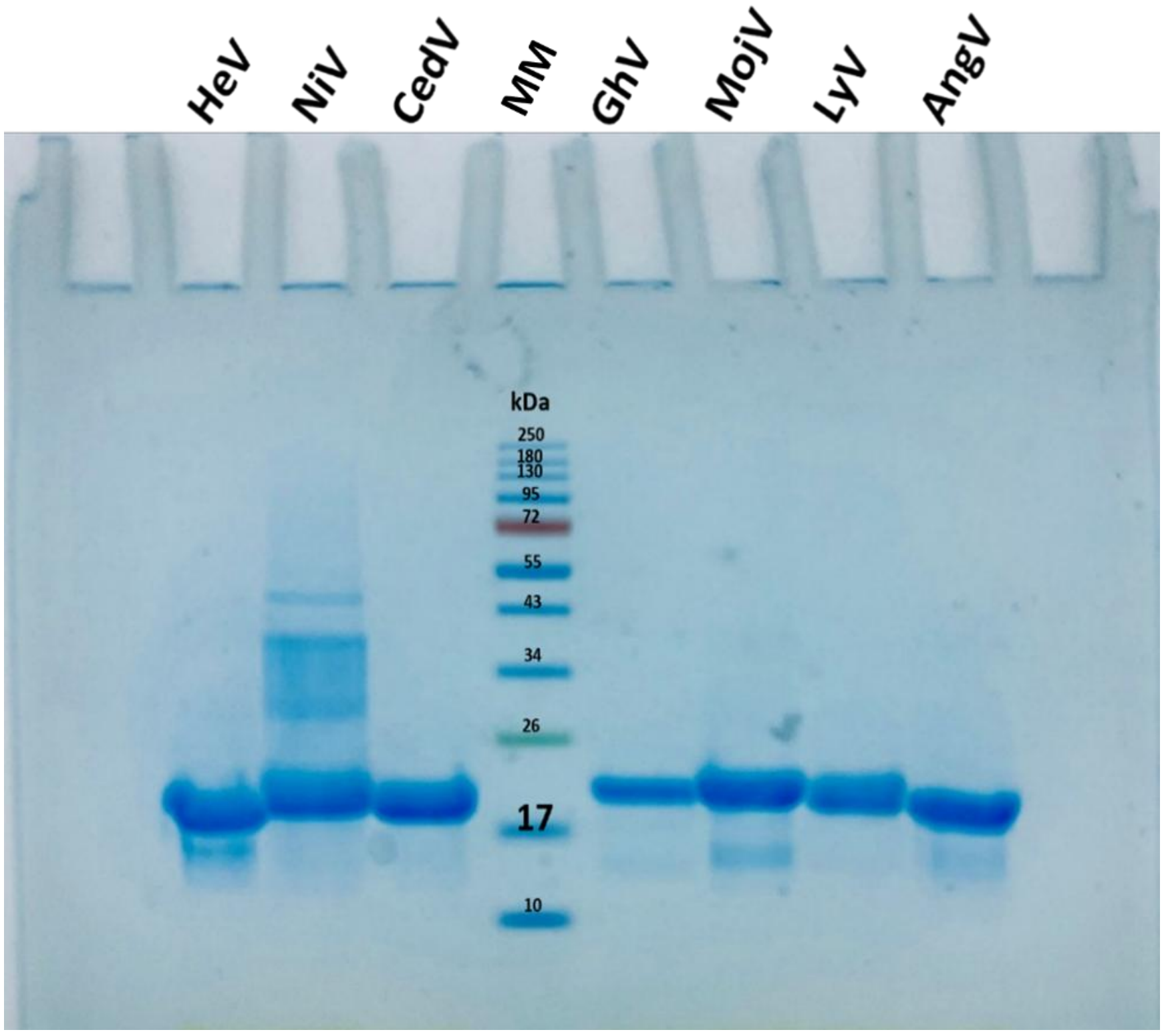

Supplementary Figure S1. SDS-PAGE (15%) analysis of the seven purified PNT1 subdomains. MM: molecular mass markers, from top to bottom: 250-10 kDa

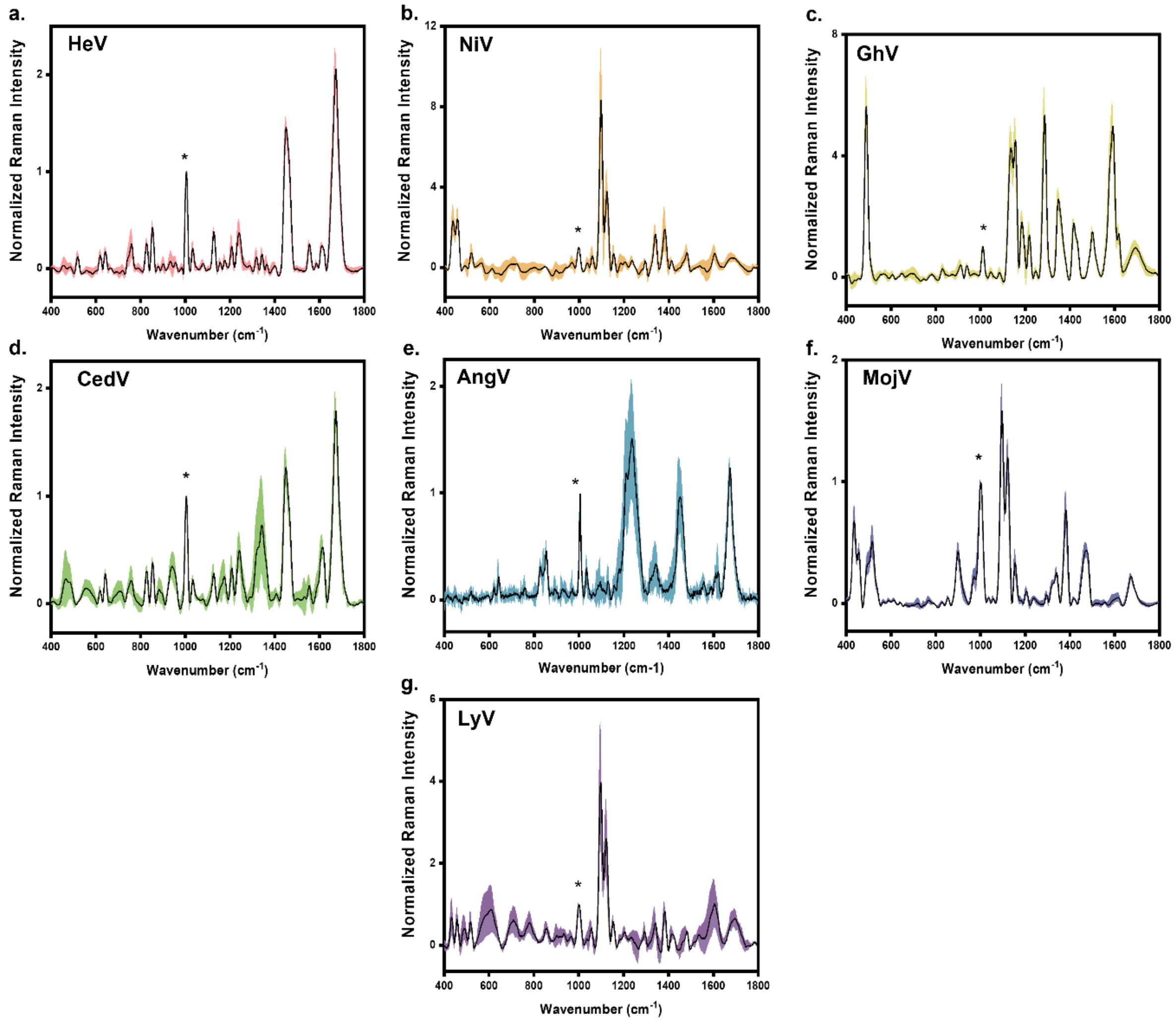

Supplementary Figure S2. Individual average Raman spectra of (a) HeV (red), (b) NiV (orange), (c) GhV (yellow) (d) CedV (green), (e) AngV (blue), (f) MojV (indigo), and (g) LyV (violet) PNT1. Spectra were recorded at 25 mW laser power, 50x objective, and 10 accumulations; n = 5. Solid lines represent the mean, whereas shaded regions represent the standard deviation. All spectra were normalized with respect to the phenylalanine ring breathing band at ~1005 cm^{-1} marked by an asterisk. See Methods for details of data acquisition, processing, and analysis.

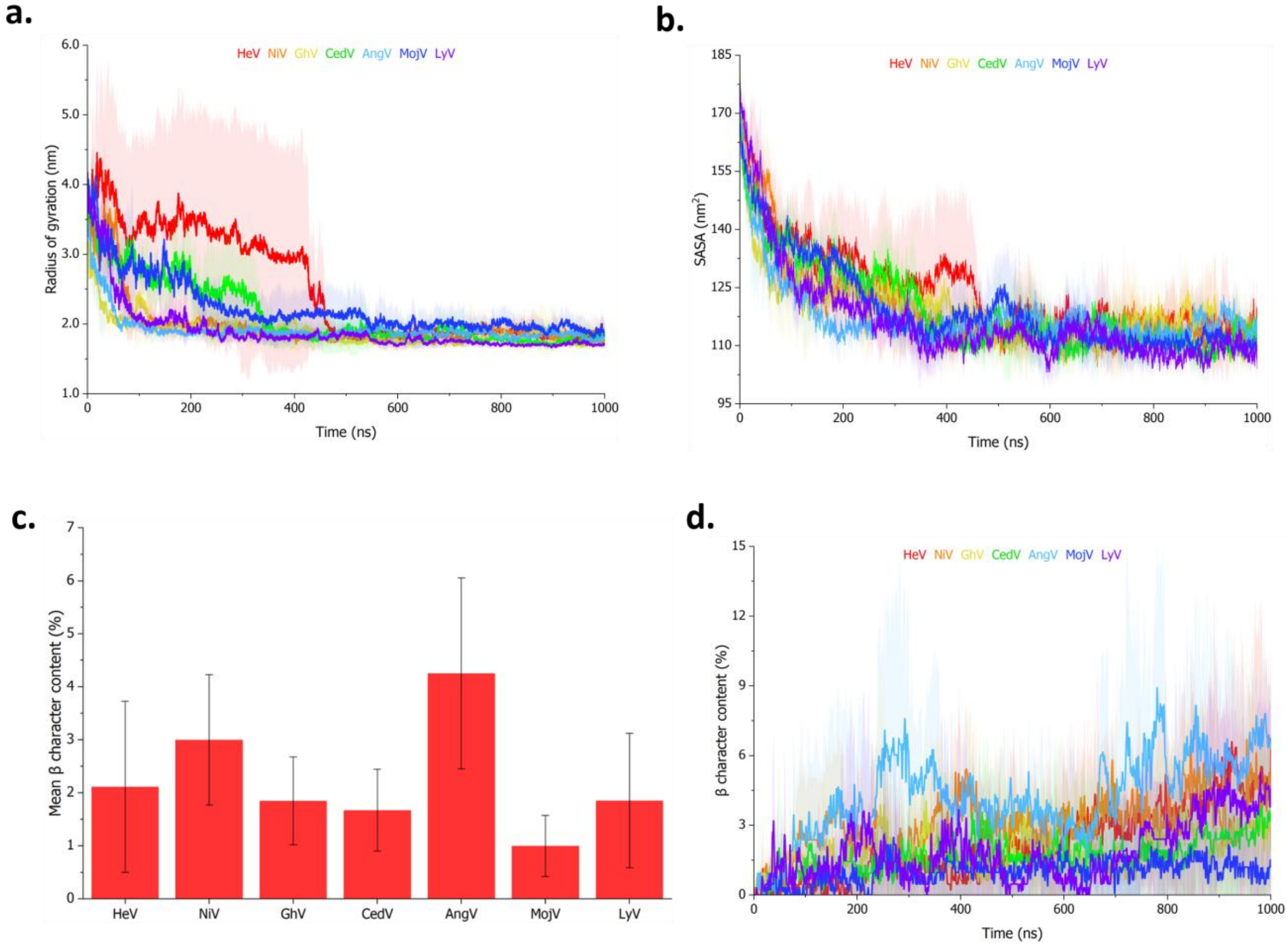

Supplementary Figure S3. (a) Time evolution of the radius of gyration (R_g) and (b) solvent-accessible surface area (SASA) for the PNT1 subdomain of HeV (red), NiV (orange), GhV (yellow), CedV (green), AngV (blue), MojV (indigo), and LyV (violet) simulated for 1 μs. Curves correspond to the average of triplicates with the standard deviation represented as a trace (shaded area) in the virus-associated color. (c) Time-averaged β character (comprising β-sheet and β-bridge) occurrence for the seven PNT1 subdomains simulated for 1 μs. Error bars represent the standard deviation of the mean β character propensity across the triplicates. (d) Time evolution of the β character (comprising β-sheet and β-bridge) content for the PNT1 subdomain of HeV (red), NiV (orange), GhV (yellow), CedV (green), AngV (blue), MojV (indigo), and LyV (violet) simulated for 1 μs. Curves correspond to the average of triplicates with the standard deviation represented as a trace (shaded area) in the virus-associated color.

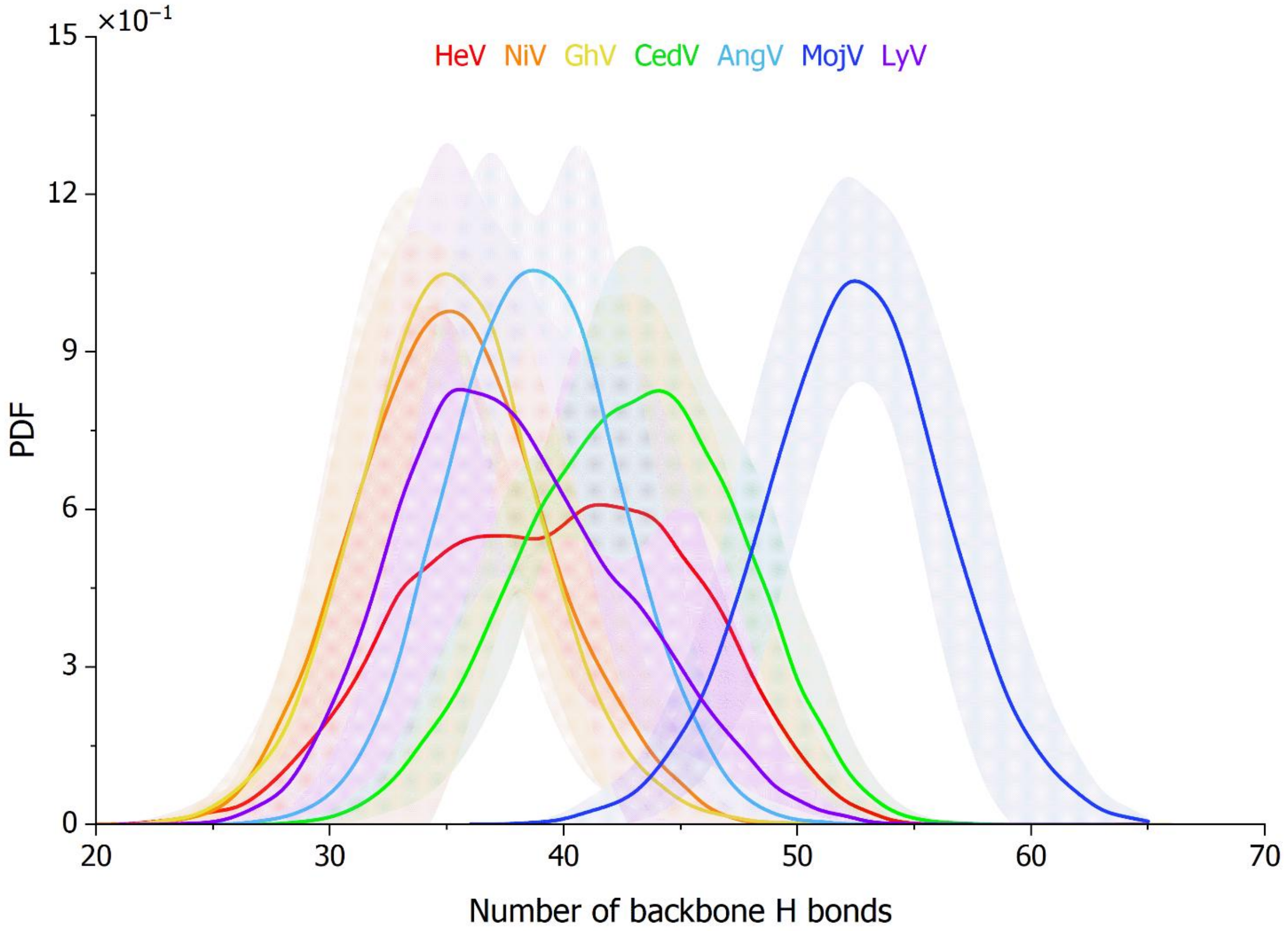

Supplementary Figure S4. Distribution of backbone H bonds, defined as a probability density function (PDF), for the PNT1 subdomain of HeV (red), NiV (orange), GhV (yellow), CedV (green), AngV (blue), MojV (indigo), and LyV (violet) simulated for 1 μs. Curves correspond to the average of triplicates with the standard deviation represented as a trace (shaded area) in the virus-associated color.

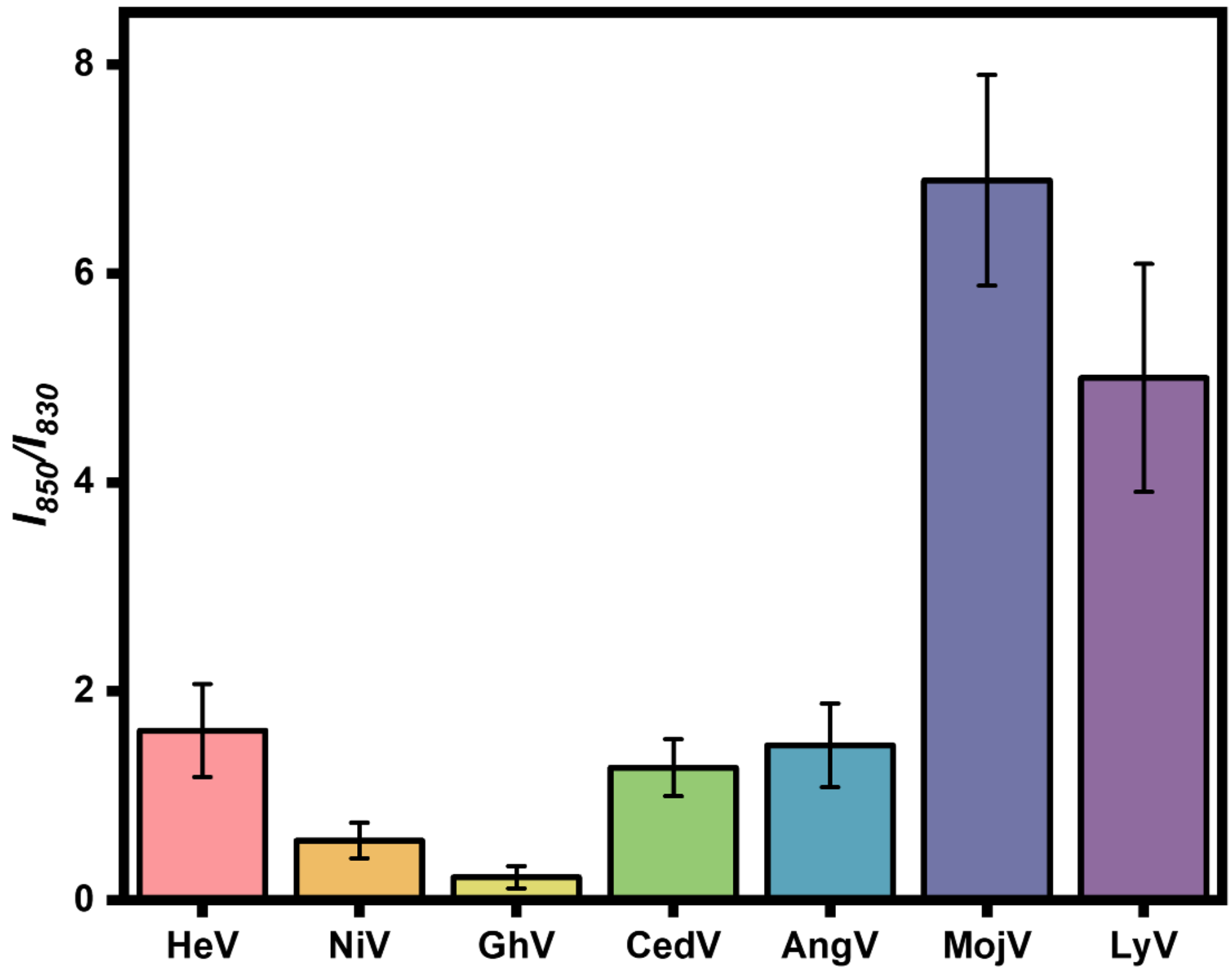

Supplementary Figure S5. Intensity ratios of the tyrosine Fermi doublet (I_{850}/I_{830}), which report on solvent-mediated hydrogen bonding propensity of the phenolic (-OH) group, for the seven PNT1 subdomains.

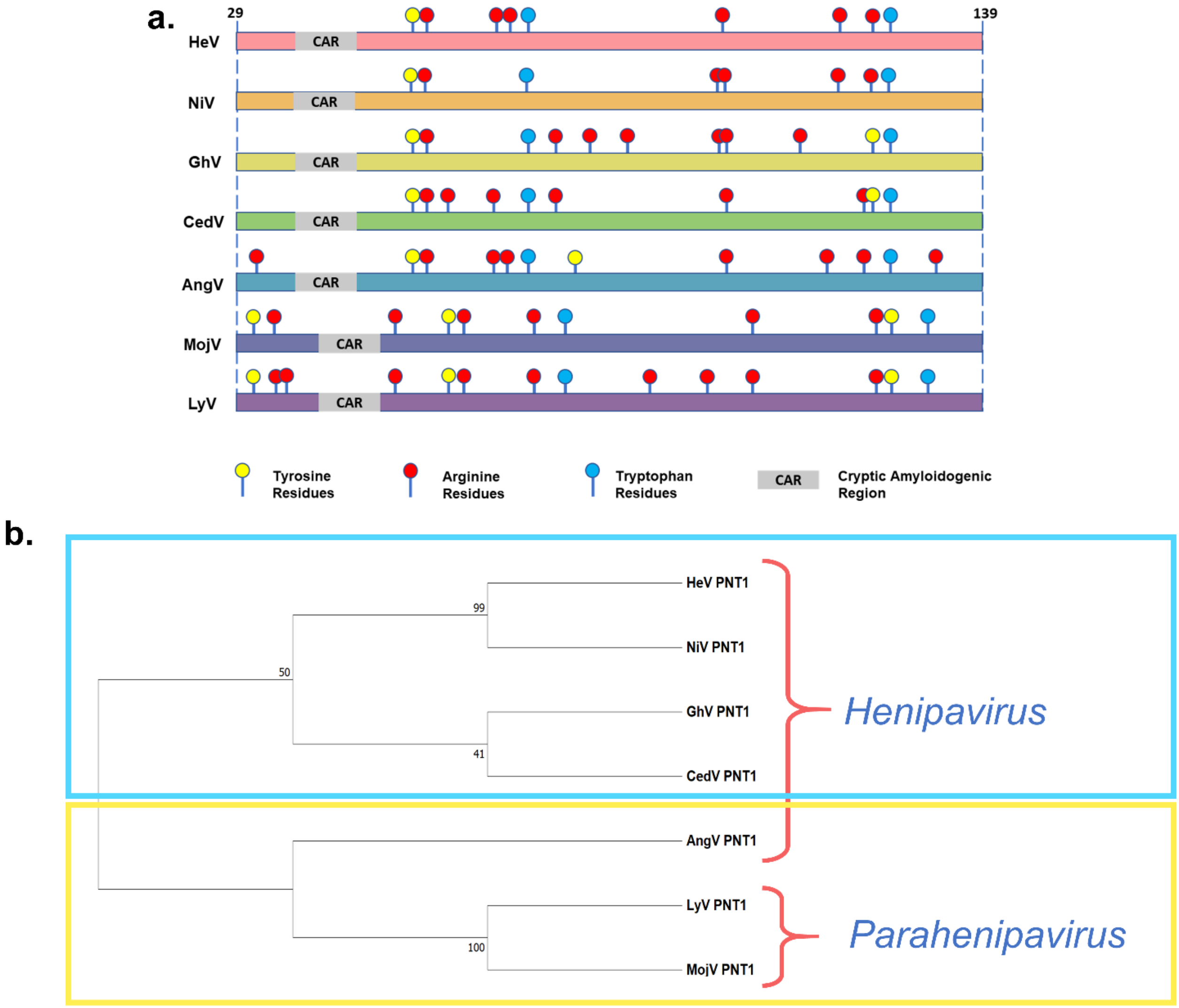

Supplementary Figure S6. (a) Schematic representation of the seven PNT1 subdomains showing the position of the conserved cryptic amyloidogenic region (CAR; grey box) together with the distribution of tyrosine (yellow), arginine (red), and tryptophan (blue) residues. The CAR is highly conserved across all homologues, whereas the spatial organization of aromatic and charged residues within the flanking intrinsically disordered regions differs substantially, indicating sequence diversification outside the conserved fibrillogenic core. (b) Phylogenetic tree of the seven PNT1 homologues. Amino acid sequences were aligned using ClustalW (Thompson *et al.*, 1994), and the phylogenetic tree was constructed using the Neighbor-Joining (NJ) method (Saitou, 1987). Branch support was assessed by bootstrap analysis with 1,000 replicates, and bootstrap values (%) are indicated at the corresponding nodes. HeV, NiV, GhV, CedV, and AngV cluster within the Henipavirus lineage, whereas LyV and MojV form a distinct evolutionary branch.

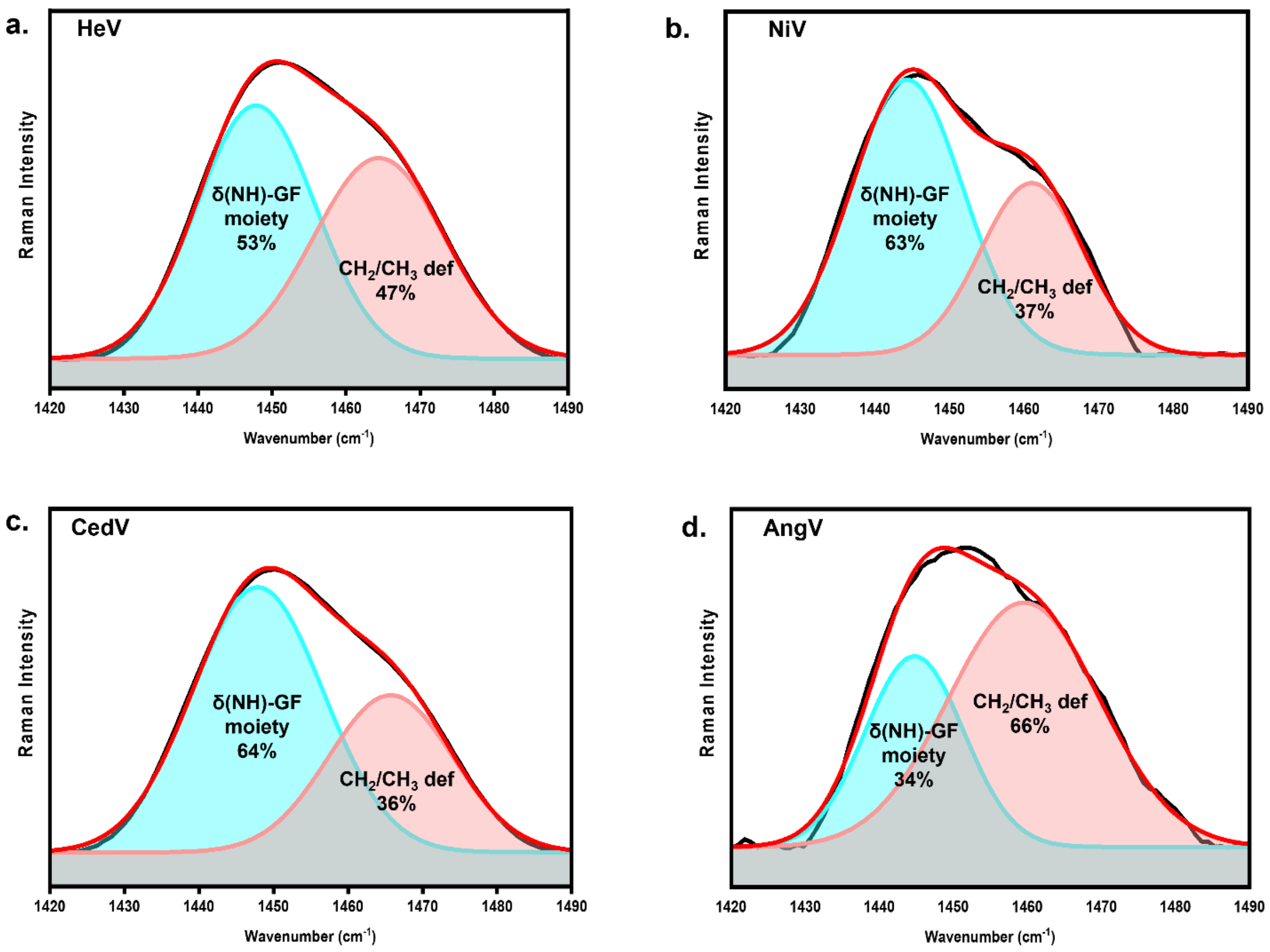

Supplementary Figure S7. Gaussian deconvolution of the region ~1420−1490 cm^{-1}. The black line represents the experimental data, averaged with n=5 datasets, and normalized with respect to Phe, while the red-colored lines represent the cumulative fit for (a) HeV, (b) NiV, (c) CedV, and (d) AngV PNT1. The cyan region represents the N–H deformations of the guanidinium fragment (GF) of arginine residues, while the salmon pink region represents the CH_2/CH_3 deformations.

Supplementary Table S1. Peak assignment of some selected modes of Raman spectra of the seven *Henipavirus* PNT1 subdomains (Tuma, 2005; Rygula *et al.*, 2013; Mensch *et al.*, 2018; Szekeres and Kneipp, 2019)

Peak Assignment§,¶	HeV	NiV	GhV	CedV	AngV	MojV	LyV
**Am I (polyproline II/random β space)**	1700	1693	1704	1700	1708	1687	1702
**Am I (alpha/β sheet and disordered)**	1672	1670	1679	1673	1678	1671	1677
**Tyr, Trp**	1610	1619	1618	1614	1621	1613	1605
**Trp**	1555	1555	1555	1555	1555	1555	1555
**δ[CH2/CH3]**	1465	1461	1432	1465	1462	1481	1423
**Asp, Glu ν_s[COO-]**	1398	1383	-	-	1390	1383	1382
**Trp**	1342	1340	1349	1342	1340	1340	1341
**Trp/δ(C_αH)**	-	-	1286	-	1293	1296	1294
**Am III (alpha), ρ(CH_α), Trp**	-	-	1286	-	-	-	1295
**Am III (disordered/random)**	1252	1250	1247	1260	1253	-	1267
**Am III (β sheet)**	1240	1237	1241	1241	1234	1236	1233
**δ(C-H), Try, Phe**	-	1182	1184	-	-	1183	1185
**Tyr, Phe, ν[C-N]**	1176	-	-	1176	1180	-	-
**Tyr, ν(Cβ-C_α-H), ν(C-C-H(R))**	-	1150	1156	1156	1157	1154	1153
**ν[C-N], ν[C-C], Pro**	1128	1125	1135	1128	1130	1122	1123
**ν[C-C], ν[C-N], ν[C-O]**	-	-	1087	-	1086	1096	1098
**ν[C-C] of skeletal of random conformation ν[CN], ν[C-O]**	-	1058	1064	-	1065	1056	1055
**Phe [δ(R(CH))]**	1034	1035	1044	1033	1032	1036	1036
**Phe [δ$R_{breathing}$)]**	1005	1006	1003	1005	1005	1002	1002
**ν[N-C_α-C], Pro, Val**	933	939	940	939	938	932	933
**Trp [δ(R+r)]**	899	892	875	882	877	898	883
**Tyr [δ(Rbreathing)]**	851	855	851	854	854	851	858
**Tyr [δ_{oop}(Rbreathing)]**	828	828	831	827	829	827	837
**Met [ν_a(C-S-C)], Val, Tyr, ν[NH], ν[CH]**	789	778	781	-		793	782
**Trp [δ(Rbreathing)]**	761	741	746	758	758	766	762
**Met [ν(C-S)]**	718	727	-	-	-	720	725
**Met [ν(C-S)], δ[CH]**	663	668	693	683		691	690
**Tyr [γ(C-C)]**	641	641	646	642	642	641	634
**δ[skeletal]**	-	519	-	523	520	515	515
**Cys[ν(S-S)]**	-	490	-	483	484	492	489
**δ[Ring], τ[Ring]**	455	457	455	464	452	455	457
**Trp**	407	-	409	409	406	407	-

§ δ, bending; τ, torsion; ν, stretching; γ, twisting; ρ, rocking; R, phenyl ring; r, pyrrole ring: a, asymmetric; s, symmetric

Supplementary Table S2. Summary of the molecular dynamics metrics, parameters, and durations for the seven simulated PNT1 subdomains from Hendra (HeV), Nipah (NiV), Ghana (GhV), Cedar (CedV), Angavokely (AngV), Mojiang (MojV), and Langya (LyV) viruses, modeled each as a single chain and solvated in explicit water in the presence of 150 mM NaCl and 0.6 M urea.

	PNT1 sub-domains						
	**HeV**	**NiV**	**GhV**	**CedV**	**AngV**	**MojV**	**LyV**
**Protein chain net charge**	–10	–10	–7	–8	–5	–9	–6
**Box volume (nm^3)**	4096	2744	2197	3375	2197	4096	3375
**Water molecules**	119683	80343	63922	109979	63978	119593	99352
**Water atoms**	359049	241029	191766	329937	191934	358779	298056
**Urea concentration (M)**	0.6	0.6	0.6	0.6	0.6	0.6	0.6
**Urea molecules**	1468	973	788	1274	761	1474	1114
**Na^+ cations**	376	254	201	340	199	375	307
**Cl^- anions**	366	244	194	332	194	366	301
**Total ionic content**	742	498	395	672	393	741	608
**Protein atoms**	2116	2138	2158	2141	2189	2141	2174
**Total atoms**	373651	251449	200623	342942	200604	373453	309750
**Ionic strength (M)**	0.15	0.15	0.15	0.15	0.15	0.15	0.15
**Minimisation (ps)**	2566	2691	2727	3002	2823	2993	3318
**NVT equilibration (ps)**	125	125	125	125	125	125	125
**Total production MD (ns)**	3*1000	3*1000	3*1000	3*1000	3*1000	3*1000	3*1000
**Total simulation time (μs)**				21			

References

Mensch,C., Bultinck,P. and Johannessen,C. (2018) *ACS Omega*, **3**, 12944–12955.

Rygula,A., Majzner,K., Marzec,K.M., Kaczor,A., Pilarczyk,M. and Baranska,M. (2013) *J Raman Spectroscopy*, **44**, 1061–1076.

Saitou,N. (1987) *Molecular Biology and Evolution*.

Szekeres,G.P. and Kneipp,J. (2019) *Frontiers in Chemistry*, **Volume 7-2019**.

Thompson,J.D., Higgins,D.G. and Gibson,T.J. (1994) *Nucl Acids Res*, **22**, 4673–4680.

Tuma,R. (2005) *J Raman Spectroscopy*, **36**, 307–319.
